# The Principle of Covariance and the Hamiltonian Formulation of General Relativity

**DOI:** 10.3390/e23020215

**Published:** 2021-02-10

**Authors:** Massimo Tessarotto, Claudio Cremaschini

**Affiliations:** 1 Department of Mathematics and Geosciences, University of Trieste, Via Valerio 12, 34127 Trieste, Italy; maxtextss@gmail.com; 2 Research Center for Theoretical Physics and Astrophysics, Institute of Physics, Silesian University in Opava, Bezručovo nám.13, CZ-74601 Opava, Czech Republic

**Keywords:** Einstein-Hilbert variational principle, Hamiltonian theory of GR, ADM Hamiltonian theory, manifest covariance, 03.50.-z, 04.20.-q, 04.20.Cv, 04.20.Fy

## Abstract

The implications of the general covariance principle for the establishment of a Hamiltonian variational formulation of classical General Relativity are addressed. The analysis is performed in the framework of the Einstein-Hilbert variational theory. Preliminarily, customary Lagrangian variational principles are reviewed, pointing out the existence of a novel variational formulation in which the class of variations remains unconstrained. As a second step, the conditions of validity of the non-manifestly covariant ADM variational theory are questioned. The main result concerns the proof of its intrinsic non-Hamiltonian character and the failure of this approach in providing a symplectic structure of space-time. In contrast, it is demonstrated that a solution reconciling the physical requirements of covariance and manifest covariance of variational theory with the existence of a classical Hamiltonian structure for the gravitational field can be reached in the framework of synchronous variational principles. Both path-integral and volume-integral realizations of the Hamilton variational principle are explicitly determined and the corresponding physical interpretations are pointed out.

## 1. Introduction

Despite being criticized in general terms by some of his contemporaries for its explicitly non-manifestly covariant approach both in classical and quantum gravity (Hawking [1], Isham [2]), nevertheless the so-called ADM Hamiltonian theory (Arnowitt, Deser and Misner [3,4]) has never been seriously challenged—possibly because of its complexity—in its mathematical and physical setup. In the following we present an attempt to do so in an exhaustive way. For this purpose, however, we shall preliminarily address the corresponding Lagrangian theory for the Einstein field equations (EFE) [5,6,7]. The review of corresponding literature approaches is useful to display the existence of a novel variational formulation whose validity is actually instrumental for the construction of a full, that is, Hamiltonian, variational theory of General Relativity (GR). Contrary to the customary Einstein-Hilbert (EH) setting, the key feature of such variational approach is that of being constraint-free, that is, subject to a so called “extremal constraint” and to be based on the identification of the variation operator, as customary in mathematical physics, in terms of the Frechet derivative. Indeed, its validity does not prevent the existence of independent (and thus unconstrained) variations of the (relevant) Lagrangian field variables. As far as ADM theory is concerned, in particular, the goal is to point out some of its most relevant critical aspects and inadequacies, which include in particular its obvious intrinsic non-manifestly covariant character. We anticipate, however, that the really crucial conclusion which is reached here is that, ADM theory does not provide, in a proper mathematical sense, a truly Hamiltonian theory of GR. Unfortunately this does not appear just a minor nuisance or an unfortunate minor accident.

The main goal of the paper thus remains that of seeking a possible way-out, that is, to determine a Hamiltonian theory of GR in a proper sense, namely in which the corresponding dynamical (i.e., Euler-Lagrange) equations are identified with ordinary differential equations and which, besides being manifestly-covariant, is also constraint-free and gauge-dependent. As we intend to show, such a theory is rooted on the classical manifestly-covariant Hamiltonian theory of GR recently developed in a series of papers [8,9,10]. For this purpose, here we intend to formulate in detail the appropriate variational principles, displaying explicitly their connection with EFE, their mutual relationship as well as their role for the Hamiltonian formulation of GR.

### Motivations and Outlook

The property of general covariance of physical laws and its explicit functional realization, namely the corresponding Principle of Manifest Covariance (PMC), represent a fundamental aspect of theoretical physics and mathematical physics, which lays at the basis of the conceptual framework of General Relativity and provides a prerequisite of consistency for the establishment of relativistic field theories (RFT). According to PMC, in particular, it should always be possible to cast the physical laws of RFT in 4-tensor form with respect to the group of local point transformations which preserve the structure of space-time [11]. The validity of such a principle represents undoubtedly a severe constraint on the admissible physical theories, requiring in many cases the need of an overhaul, or even a complete reformulation, of existing theories. In fact it provides strict guidelines and recipes on the formal representation of mathematical relationships holding among the same laws, but also encodes a deeper meaning related to the admissible realization (and possible universal character) of the relevant equations, physical parameters, classical or quantum phase-functions and observables. In this sense, general covariance affects both the classical and quantum description of continuum fields in RFT, for the search respectively of appropriate variational (i.e., Lagrangian and Hamiltonian) formulations for classical field theories and of canonical quantization methods for the corresponding quantum field theories.

The general covariance principle must therefore be interpreted as a paramount guiding principle for the consistent variational formulation of classical equations of General Relativity and the establishment of a quantization approach yielding a consistent covariant quantum gravity theory, possibly including the combined effect due to classical or quantum source fields, like the electromagnetic or scalar fields to be described on the same ground.

The subject of this paper is the investigation of the validity of the principle of covariance for the variational formulation of classical General Relativity and its implications for the establishment of a Hamiltonian theory for the Einstein field equations (EFE). The focus here is on the conceptual and mathematical meaning of the principle of general and manifest covariance in classical General Relativity, with particular attention to the identification of the Hamiltonian structure underlying the Einstein field equations and the validity of corresponding Hamiltonian variational principles. This target requires necessarily a critical analysis of the conditions of validity of the Hamiltonian formulation of GR available in existing literature, which is realized by the well-known 3 + 1 ADM theory, in relation to the variational Lagrangian formulation of GR, as well as the establishment of the problematic limitations of such approach in connection with the principles of covariance and manifest covariance. More precisely, three main issues arise in this procedure, which pertain:

(1) A critical analysis of the Einstein-Hilbert Lagrangian variational principle that determines EFE in manifestly-covariant form as extremal Euler-Lagrange equations, when the corresponding Lagrangian function is identified with the Ricci curvature 4-scalar in which the variational field variable is represented by the space-time metric tensor $g_{\mu \nu }$. In fact, the notorious difficulty which characterizes this approach to the Lagrangian formulation of GR arises due to the non-canonical structure of the E-H Lagrangian function that carries second-order partial derivatives of the variational metric tensor contained in the Ricci tensor. As such, distinctive realizations can be envisaged for the same variational principle, differing for the functional dependence of the Ricci curvature tensor on the variational fields and the way in which its variation is treated. The goal of the investigation is to show that the approaches available in the literature can be classified as asynchronous boundary-constrained variational principles for the EH theory. In this framework, the contributions generated by variations of the metric tensor carried by the Ricci tensor cannot contribute to the extremal equations if the latter are to be identified with EFE. Therefore, this necessarily requires to set up appropriate boundary constraints that warrant the drop of such undesirable variational terms. In contrast, a novel formulation is pointed out, which realizes an unconstrained form free of previous boundary constraints and that exhibits also the remarkable features of restoring the canonical form for the Lagrangian function (which depends on field variable and at most on its first-order derivative), of satisfying the principle of covariance and of yielding EFE as extremal equations.

(2) The investigation of the ADM variational formulation (briefly ADM theory) obtained by implementing a 3 + 1 splitting of space-time in terms of so-called ADM field variables and its relationship with the Einstein-Hilbert variational approach in connection with the Hamiltonian formulation of GR. A crucial aspect of ADM theory lies, in fact, in the violation of the principle of manifest covariance due to the peculiar choice of reference system in which time and space coordinates are no longer treated on equal footing, and in particular where a suitable coordinate-time is singled out to play the role of dynamical (i.e., evolution) parameter for the theory. In the literature this peculiar aspect is regarded as an intrinsic property of Hamiltonian theories for continuum fields, in contrast to the manifestly-covariant form of corresponding Lagrangian formulations. For this reason, in the scientific community the implementation of ADM theory in the E-H variational principle is recognized as the viable route that leads to the definition of a 3-dimensional constrained Hamiltonian representation of EFE. However, based on the results pointed out in (1), a deeper insightful critical analysis of the Hamiltonian character of the Einstein equations is required in order to question the foundation of the subject. The strategy that is followed consists in expressing both the boundary-constrained and unconstrained Lagrangian principles in terms of the corresponding non-manifestly covariant ADM frameworks and then to require the simultaneous validity of the resulting ADM variational equations and their equivalence with EFE. This yields the main result of the work, which concerns the proof of the non-Hamiltonian character of ADM theory and the failure of this approach in providing a symplectic structure of space-time. Contrary to the common belief, it is not sufficient to implement a suitable coordinate system where a time variable is distinguished from space variables in order to obtain a Hamiltonian field theory. Indeed, the ADM Hamiltonian theory does not represent an intrinsic Hamiltonian structure of space-time, but only an apparent one. The reason is that also the ADM formulation remains rooted on the non-canonical structure of the E-H Lagrangian, and it still carries second-order derivatives of the generalized coordinate field, an aspect which is sufficient to prevent the definition of canonical Hamiltonian theory.

(3) The search and construction of a new Hamiltonian representation of EFE which can overcome the conceptual limitations of the ADM setting and be simultaneously globally valid, unconstrained and manifestly covariant. In particular, it is proved that a solution reconciling the physical requirements of covariance and manifest covariance of variational theory with the existence of a classical Hamiltonian structure for the gravitational field corresponding to a true symplectic structure of space-time can be effectively reached in the framework of synchronous variational principles, based on the outcomes earlier provided by Refs. [8,9,10]. In order to afford a straightforward comparison with the ADM setting, the parametrization in terms of an invariant proper-time parameter is first introduced. This allows in turn to construct synchronous Hamiltonian variational principles expressed either by path-integral or volume-integral realizations. Remarkably, both representations yield manifestly-covariant Hamilton equations in canonical form, namely expressed as evolution equations in terms of the proper-time dynamical parameter, which under prescription of suitable initial conditions for the canonical state recover identically EFE. The novelty of the theory lies also in the corresponding physical interpretation that is inferred about the role of the Ricci 4-scalar in the EH Lagrangian function, which motivates and supports in turn this kind of approach. In fact, consistent with the principle of manifest covariance, within the framework of the synchronous Hamiltonian variational principle the latter should be understood as a potential term expressing the coupling between the variational metric tensor and the Ricci tensor carrying information about the geometry of the background space-time.

The subject of the present investigation summarized in points (1)–(3) listed above can establish a reference framework for the theoretical foundations of GR Lagrangian and Hamiltonian variational formulations. Conceptual implications of the theory involve both classical GR as well as quantum gravity, namely perspective covariant canonical quantization approaches for the gravitational field based on the same Hamiltonian theory.

## 2. Asynchronous Variational Approaches

The subject of the investigation is therefore centered on the crucial relationship between the covariance principle and the Hamiltonian theory of GR.

To start with we stress that a key issue concerns couching the theory on the appropriate mathematical framework, that is, based on a suitable prescription of the related functional setting and the notion of functional variation. For this purpose a preliminary brief survey is appropriate regarding some of the popular variational principles for GR equations that are customarily adopted in the literature. The reason, as anticipated above, is to point out a realization of the Lagrangian variational principle (for EFE) which, besides being manifestly covariant, is also unconstrained. Such a feature, which departs from previous constrained formulations found in the literature, is of crucial importance. In fact, it turns out to be an essential ingredient for the establishment of a corresponding constraint-free Hamiltonian variational formulation (see Section 4).

The review presented here pertains in particular to the Einstein-Hilbert (EH) Lagrangian variational approach (referred to for brevity as EH theory), originally developed by Einstein himself to determine the actual form of EFE. For this purpose, we consider first the so-called asynchronous variational principle. This case occurs when the functional setting is prescribed so that the 4-scalar volume element associated with the variational functional depends functionally on suitable real and symmetric fields $g\left(r\right)\equiv \left\{g_{\mu \nu }\right\}\equiv \left\{g^{\mu \nu }\right\}$. The latter are then considered variational, that is, still undetermined 4-tensors. The relevant variational functional which occurs in EH theory is the so-called EH action functional [5]. In the general case of source fields, that is, non-vacuum conditions, this is identified with the 4-scalar functional

(1) $$S_{EH}\left(g\left(r\right)\right)=\int_{Q^{4}}d^{4}r\sqrt{-\left|g\right|}\left(L_{EH}\left(g\right)+L_{F}\left(g\right)\right),$$

where the argument g=g(r) denotes the functional dependence in terms of the variational field, while

(2) $$d\Omega \equiv d^{4}r\sqrt{-\left|g\right|}$$

is the invariant 4-scalar volume element of the Riemann spacetime $\left\{Q^{4},g\left(r\right)\right\}$ and $\left|g\right|$ under the square root denotes the determinant of g(r). Furthermore, $L_{EH}$ and $L_{F}$, both assumed as 4-scalars, identify the Einstein-Hilbert Lagrangian function of the gravitational field and the Lagrangian of possible additional fields, to be properly specified. By definition, here the function $L_{EH}$ represents the vacuum contribution

(3) $$L_{EH}=g^{\mu \nu }R_{\mu \nu }-2\Lambda ,$$

where in standard notation Λ denotes the cosmological constant, while $R_{\mu \nu }$ is the variational Ricci tensor which is considered function of the same variational tensor $g_{\mu \nu }$ by means of the Christoffel symbols. As such it carries the second partial derivatives of $g_{\mu \nu }$ with respect to the 4-position $r^{\mu }$. Notice that in this framework the variational tensor field $g\left(r\right)\equiv \left\{g_{\mu \nu }\right\}$ is also a metric tensor associated with the spacetime $\left\{Q^{4},g\right\}$ ($Q^{4}\equiv R^{4}$), so that it raises and lowers tensor indices and hence must satisfy suitable orthogonality conditions. This means that its functional setting, including the same conditions, is constrained in the sense that it is necessarily of the form

(4) $${\left\{g\right\}}_{C}\equiv \left\{\left.g\left(r\right)\equiv g_{extr}\left(r\right)+\delta g\left(r\right)\in C^{2}\left(Q^{4}\right)\right|g_{\mu \nu }g^{\mu k}=\delta_{\nu }^{k}\right\},$$

with $Q^{4}\equiv R^{4}$ and ${\left\{g\right\}}_{C}$ denoting the functional class of “normalized” varied functions g(r). Furthermore, here the 10 covariant and counter-variant components of symmetric tensor field *g*, namely respectively $g_{\mu \nu }$ and $g^{\mu \nu }$, are considered linearly independent, despite being required to fulfill the orthogonality constraints

(5) $$g_{\mu \nu }g^{\mu k}=\delta_{\nu }^{k},$$

which imply in turn the “normalization” condition $g_{\mu \nu }g^{\mu \nu }=4$. In addition, each varied function g(r) is assumed of the form

(6) $$g\left(r\right)\equiv g_{extr}\left(r\right)+\delta g\left(r\right),$$

$g_{extr}\equiv \left\{g_{{}_{extr},\mu \nu }\left(r\right)\right\}\equiv \left\{g_{{}_{extr}}^{\mu \nu }\left(r\right)\right\}$ representing a suitably defined extremal real and symmetric tensor field (see below) and $\delta g\left(r\right)\equiv \left\{\delta g^{\mu \nu }\right\}\equiv \left\{\delta g_{\mu \nu }\right\}$ a real and symmetric variation tensor field. We stress here that in the framework of EH theory each $g\left(r\right)\equiv \left\{g_{\mu \nu }\left(r\right)\right\}\equiv \left\{g^{\mu \nu }\left(r\right)\right\}$ is regarded as a metric tensor field which raises and lowers tensor indices and for each $g\left(r\right)\equiv \left\{g_{\mu \nu }\left(r\right)\right\}\equiv \left\{g^{\mu \nu }\left(r\right)\right\}$ its countervariant components $\left\{g^{\mu \nu }\right\}$ are by construction prescribed so that the orthogonality conditions (5) are identically satisfied. In addition, the variations $\delta g_{\mu \nu }$ are regarded as linearly independent and possibly subject to suitable boundary conditions (to be applied on appropriate boundaries). Finally, in the same setting the three quantities $\sqrt{-\left|g\right|}$, $g^{\mu \nu }$ and $R_{\mu \nu }$ are all considered as variational in Equation (1), although $\sqrt{-\left|g\right|}$ is not by itself a 4-tensor.

### 2.1. The Einstein-Hilbert Lagrangian Variational Principle

Given these premises, we first address in particular the Einstein-Hilbert (EH) variational principle. In this case the extremal field $g_{extr}\left(r\right)$, which enters Equation (6), is identified with the solution of the Einstein field equations and therefore for better clarity is denoted here with the symbol

(7) $$g_{extr}\left(r\right)\equiv \hat{g}\left(r\right)=\left\{{\hat{g}}_{\mu \nu }\left(r\right)\right\}.$$

The tensor field $\hat{g}\left(r\right)$ will be henceforth referred to as “background” metric tensor field. Then, the EH variational principle (or EH action principle) is obtained by prescribing that for arbitrary variations δg(r) it occurs that:

(8) $${\left.\delta S_{EH}\left(g\right)\right|}_{g=\hat{g}\left(r\right)}=0,$$

with the symbol δ denoting, as usual, the variation operator. Notice that here the precise mathematical definitions matter. In fact, a (possible) departure from previous literature lies in the definition of the variation operator δ adopted here and the prescription of its functional setting, which in case (8) is identified with ${\left\{g\right\}}_{C}$ (see, e.g., Equation (4)). Thus, according to the standard definition available in the mathematical literature [12], for any real smooth function or functional F(g(r)) its functional variation is identified with its Frechet derivative. The definition is based on the representation of a generic varied function g(r) which, thanks to Equation (7), is given now in terms of

(9) $$g\left(r\right)=\hat{g}\left(r\right)+\delta g\left(r\right).$$

Furthermore, for definiteness, we denote by g(r,α) the related parametrized varied function

(10) $$g\left(r,\alpha \right)=\hat{g}\left(r\right)+\alpha \delta g\left(r\right),$$

with $\alpha \in \left[-1,1\right]$ being a real 4-scalar parameter to be assumed independent of *r*. Then, the Frechet derivative of the function (or functional) F(g(r)) is given by

(11) $$\delta F\left(g\left(r\right)\right)\equiv {\left.\frac{d}{d\alpha }F\left(\hat{g}\left(r\right)+\alpha \delta g\left(r\right)\right)\right|}_{\alpha =0}.$$

The obvious implication is therefore that the variation of the Ricci tensor $R_{\mu \nu }$ when it is considered as a function (or functional) of the variational tensor field g(r), is therefore generally non-zero (for non-vanishing variation δg(r)) because the dependence of the Christoffel symbols $\Gamma_{il}^{m}$ in terms of the variational field g(r) implies that

(12) $$\delta \Gamma_{il}^{m}\left(g\left(r\right)\right)\equiv {\left.\frac{d}{d\alpha }\Gamma_{il}^{m}\left(\hat{g}\left(r\right)+\alpha \delta g\left(r\right)\right)\right|}_{\alpha =0},$$

which is therefore different from zero. We stress, on the other hand, that by construction $\delta \hat{g}\left(r\right)=0$, so that if one assumes $\Gamma_{il}^{m}=\Gamma_{il}^{m}\left(\hat{g}\left(r\right)\right)$, then identically

(13) $$\delta \Gamma_{il}^{m}\left(\hat{g}\left(r\right)\right)\equiv 0.$$

Notice, however, that this prescription differs from the so-called Palatini approach [13] in which the Christoffel symbols $\Gamma_{il}^{m}$ are considered independent variables and as such can therefore in principle be required to satisfy the constraint requirement

(14) $$\delta \Gamma_{il}^{m}=0$$

if the variation δ is performed only with respect to δg(r).

We stress that in Equation (8) the tensor field $\hat{g}\left(r\right)$ is prescribed in such a way that the same equation is satisfied identically for arbitrary variations δg(r). The variation of all relevant functional or functions can similarly be defined. Thus, while the variation of $\hat{g}\left(r\right)$ vanishes identically, being independent of α, so that $\delta \hat{g}\left(r\right)={\left.\frac{d}{d\alpha }\hat{g}\left(r\right)\right|}_{\alpha =0}=0$, by definition $\delta g={\left.\frac{d}{d\alpha }g\left(r,\alpha \right)\right|}_{\alpha =0}$. Hence, the operator δ defined in this way does not preserve the 4-scalar volume element dΩ, since

(15) $$\delta d\Omega \equiv d^{4}r\delta \sqrt{-\left|g\right|}\neq 0,$$

and for this reason is referred to as *asynchronous variational operator*. Because of the consequent explicit variational contribution which arises due to the non-constant volume element, and in analogy with the terminology adopted in classical mechanics, the EH variational principle (8) is thus denoted as *asynchronous variational principle.*

Its implications are in principle straightforward. In fact, by identifying the *variational Lagrangian density* $L_{var}\equiv L_{var}\left(g\right)$ with

(16) $$L_{var}\left(g\right)=\sqrt{-\left|g\right|}\left(L_{EH}\left(g\right)+L_{F}\left(g\right)\right),$$

the variational principle (8) yields the corresponding Euler-Lagrange equations, which in symbolic form (i.e., expressed in terms of the functional derivative with respect to $g^{\mu \nu }$, or equivalently $g_{\mu \nu }$) take the form

(17) $${\left.\frac{\delta S_{EH}}{\delta g^{\mu \nu }}\right|}_{g=\hat{g}\left(r\right)}\equiv {\left.\frac{\partial L_{var}\left(g\right)}{\partial g^{\mu \nu }}\right|}_{g=\hat{g}\left(r\right)}=0.$$

Notice that here the solution ($\hat{g}\left(r\right)$) of the Euler-Lagrange Equation (17) necessarily coincides with the extremal value of the variational tensor field *g*, that is, such that Equation (8) is satisfied for arbitrary variations δg(r) belonging to the functional class ${\left\{g\right\}}_{C}$. Nevertheless, the precise evaluation of the Frechet derivative (11) and its related functional derivative, that is, Equation (17), are not straightforward, the reason being that the Ricci tensor appearing in $L_{EH}\left(g\right)$ contains second-order partial derivatives of g(r).

### 2.2. Boundary-Constrained Variational Principle of EH Theory

In order to tackle such a problem it is convenient to distinguish in the EH variational principle (8) two variational contributions, by letting

(18) $$\delta S_{EH}\left(g\right)={\left(\delta S_{EH}\left(g\right)\right)}_{\mathrm{expl}}+{\left(\delta S_{EH}\left(g\right)\right)}_{\mathrm{impl}},$$

with ${\left(\delta S_{EH}\left(g\right)\right)}_{\mathrm{expl}}$ and ${\left(\delta S_{EH}\left(g\right)\right)}_{\mathrm{impl}}$ denoting respectively the explicit and implicit variations of the action functional $S_{EH}\left(g\right)$. The first one is obtained by varying $S_{EH}\left(g\right)$ while keeping the Ricci tensor constant, while the second one contains the variation of the Ricci tensor only (see Equations (3) and (6) below). Thus, in the vacuum case with non-vanishing cosmological constant the first contribution contains the explicit contributions coming respectively: (a) from the variation of the determinant entering the 4-scalar volume element (2), that is, yielding its *explicit asynchronous variation* $\delta d\Omega =d^{4}x\delta \sqrt{-\left|g\right|}$, where the identity

(19) $$\delta \sqrt{-\left|g\right|}=-\frac{1}{2}g_{\mu \nu }\delta g^{\mu \nu }$$

holds; (b) from the explicit contribution carried by $g^{\mu \nu }$ in $L_{EH}$ and $L_{F}$ (with $L_{F}$ being assumed independent of $R_{\mu \nu }$). As a consequence:

(20) $$\begin{array}{ccc} {\left(\delta S_{EH}\left(g\right)\right)}_{\mathrm{expl}} & = & \int_{Q^{4}}d^{4}r\left[\left(\delta \sqrt{-\left|g\right|}\right)\left[g^{\mu \nu }R_{\mu \nu }-2\Lambda \right]+\sqrt{-\left|g\right|}\left(\delta g^{\mu \nu }\right)R_{\mu \nu }\right]\\ & & +\delta \int_{Q^{4}}d^{4}r\sqrt{-\left|g\right|}L_{F}\left(g\right). \end{array}$$

Direct evaluation of the various terms on the rhs of Equation (3) yields

(21) $$\begin{array}{ccc} {\left(\delta S_{EH}\left(g\right)\right)}_{\mathrm{expl}} & = & \int_{Q^{4}}d^{4}r\sqrt{-\left|g\right|}\left[R_{\mu \nu }-\left(\frac{1}{2}R-\Lambda \right)g_{\mu \nu }\right]\delta g^{\mu \nu }\\ & & -\int_{Q^{4}}d^{4}r\sqrt{-\left|g\right|}\delta g^{\mu \nu }\kappa T_{\mu \nu }, \end{array}$$

which implies

(22) $$R_{\mu \nu }-\left(\frac{1}{2}R-\Lambda \right)g_{\mu \nu }=\kappa T_{\mu \nu },$$

namely, the general form of the non-vacuum Einstein field equations (EFE) for the unknown tensor field $g_{\mu \nu }\left(r\right)$. Here the notation is standard. Thus, $R_{\mu \nu }$ and $R=g^{\mu \nu }R_{\mu \nu }$ are respectively the Ricci 4-tensor and Ricci 4-scalar, Λ is the 4-scalar cosmological constant, while $T_{\mu \nu }$ is the stress-energy tensor associated with the external source fields described by the external-field Lagrangian density $L_{F}\left(g\right)$. Finally, κ is the universal constant $\kappa =8\pi G/c^{4}$, while *G* is the Newtonian constant of gravitation with *c* being the speed of light in vacuum. The 4-tensor Equation (5) represents a second-order, intrinsically non-Hamiltonian PDE, whose solution will be denoted here with the symbol $\hat{g}\left(r\right)=\left\{{\hat{g}}_{\mu \nu }\left(r\right)\right\}$.

Given the result expressed by Equation (5) one therefore expects that the remaining contributions should vanish identically. These are carried by the *implicit variation* ${\left(\delta S_{EH}\left(g\right)\right)}_{\mathrm{impl}}$, obtained by varying $S_{EH}\left(g\right)$ with respect to the Ricci tensor only, namely:

(23) $${\left(\delta S_{EH}\left(g\right)\right)}_{\mathrm{impl}}=\int_{Q^{4}}d^{4}r\sqrt{-\left|g\right|}g^{\mu \nu }\delta R_{\mu \nu }.$$

The obvious implication is therefore that the variational functional constraint condition

(24) $${\left(\delta S_{EH}\left(g\right)\right)}_{\mathrm{impl}}=0$$

must necessarily hold in order to recover exactly EFE. As a consequence, the EH variational principle in Equation (8) must be considered as a constrained one, the constraint (24) realizing effectively a *boundary-constrained EH variational principle*. The peculiar difficulty that arises when trying to satisfy the variational constraint condition (24), however, is due to the fact that, as anticipated, the Ricci tensor contains second-order partial derivatives of the metric tensor, so that on the whole the variational Lagrangian is not written in standard form and one obtains that the implicit term still carries first-order derivatives of the generalized coordinate field $g^{\mu \nu }$. In fact, let us recall the definition of the Ricci scalar and tensor (*R* and $R_{\mu \nu }$)

(25) $$R=g^{\mu \nu }R_{\mu \nu }=G+g^{\mu \nu }\left[\frac{\partial \Gamma_{\mu \nu }^{l}}{\partial r^{l}}-\frac{\partial \Gamma_{\mu l}^{l}}{\partial r^{\nu }}\right],$$

where *G* is a function of $g^{\mu \nu }$ of the form

(26) $$\begin{array}{ccc} G & = & H_{\mu \nu }\left(\Gamma \right)g^{\mu \nu },\\ H_{\mu \nu }\left(\Gamma \right) & \equiv & \Gamma_{il}^{m}\Gamma_{km}^{l}-\Gamma_{ik}^{l}\Gamma_{lm}^{m}, \end{array}$$

and $\Gamma_{il}^{m}$ are the Christoffel symbols prescribed with respect to $g^{\mu \nu }$. Since by construction $\nabla_{\alpha }g^{\mu \nu }\equiv 0$, explicit calculation gives

(27) $$\int_{Q^{4}}d^{4}r\sqrt{-\left|g\right|}R=\int_{Q^{4}}d^{4}r\sqrt{-\left|g\right|}G+\int_{Q^{4}}d^{4}r\sqrt{-\left|g\right|}\nabla_{\alpha }\left[g^{\beta k}\Gamma_{\beta k}^{\alpha }-g^{\beta \alpha }\Gamma_{\beta k}^{k}\right].$$

As a consequence, it follows that in order to satisfy the constraint (24) it should be identically:

(28) $${\left(\delta S_{EH}\left(g\right)\right)}_{\mathrm{impl}}=\int_{Q^{4}}d^{4}r\sqrt{-\left|g\right|}g^{\mu \nu }\delta H_{\mu \nu }\left(\Gamma \right)+\int_{Q^{4}}d^{4}r\sqrt{-\left|g\right|}\nabla_{\alpha }w^{\alpha }=0,$$

with $w^{\alpha }$ denoting the 4-vector

(29) $$w^{\alpha }=g^{\beta k}\delta \Gamma_{\beta k}^{\alpha }-g^{\beta \alpha }\delta \Gamma_{\beta k}^{k}.$$

Thus, in particular, the last integral thanks to the divergence theorem reduces to

(30) $$\int_{Q^{4}}d^{4}r\sqrt{-\left|g\right|}\nabla_{\alpha }w^{\alpha }=\int_{\partial Q^{4}}d\sigma n_{\alpha }w^{\alpha },$$

with $n_{\alpha }$ denoting the inward unit tensor locally orthogonal to the surface $\partial Q^{4}$ of the boundary $Q^{4}$. In the literature various possible tentative solutions have been proposed to satisfy the constraint Equation (28).

*Choice A*—The first approach is the one proposed by Einstein himself [11] (see also Landau-Lifschitz [5]). The claim is that it should be sufficient to impose that on the boundary $\partial Q^{4}$ of integration domain the variations $\delta \Gamma_{ik}^{l}$ and $\delta \Gamma_{lk}^{k}$ are prescribed in such a way that the 4-vector $w^{\alpha }$ is set identically zero, namely

(31) $$w^{\alpha }{|}_{\partial Q^{4}}=0.$$

In fact, according to the same claim, the contribution of the volume integral in the first term on the rhs of Equation (28) should be regarded as null being evaluated in the local frame in which the spacetime is locally flat, so that the tensor function $H_{\mu \nu }\left(\Gamma \right)$ should remain constant with respect to arbitrary variations, so that in a locally flat system

(32) $$\int_{Q^{4}}d^{4}r\sqrt{-\left|g\right|}g^{\mu \nu }\delta H_{\mu \nu }\left(\Gamma \right)=0.$$

A criticism arises on this approach. We first notice that the boundary condition (31) is perfectly admissible. It can be viewed, in fact, as a gauge condition to the variational Lagrangian density $L_{var}\left(g\right)$ (16). Indeed it is always possible to add to $L_{var}\left(g\right)$ a suitable boundary term contribution which cancels exactly a possible non-vanishing surface boundary term $w^{\alpha }{|}_{\partial Q^{4}}\neq 0$ which might be carried by Lagrangian density.

The argument leading to the proof of Equation (32) is instead of dubious validity. The objection is as follows. Validity of Equation (32) for arbitrary variations δg(r) belonging to ${\left\{g(r)\right\}}_{C}$ requires, in turn, that the Christoffel symbols should be almost everywhere such that

(33) $$\delta H_{\mu \nu }\left(\Gamma \right)=0.$$

However, for a prescribed metric tensor $g\left(r\right)=\hat{g}\left(r\right)+\delta g\left(r\right)$ and a bounded set $Q^{4}$, the approximate local constraint condition

(34) $$\delta H_{\mu \nu }\left(\Gamma \right)=0+O\left(\varepsilon^{\gamma }\right)$$

can be fulfilled at most on a countable set of covering ε-neighborhoods $V_{\varepsilon }\left(r_{i}\right)$ of $r_{i}\in$$Q^{4}$, with radius ε (being ε a suitable infinitesimal and γ a suitable constant >1). Nevertheless, such a choice of the coordinate system does not warrant that the same requirement (34) is satisfied for arbitrary variations δg(r) belonging to ${\left\{g(r)\right\}}_{C}$. This implies that Equation (34) cannot be realized by a local point transformation which preserves the differential manifold $\left\{Q^{4},g\left(r\right)\right\}$.

As a note added in proof we notice that it is possible to show [14] that a diffeomorphism which maps in each other almost everywhere a curved spacetime $\left\{Q^{4},g\left(r\right)\right\}$ on the flat Minkowski spacetime $\left\{Q^{4},\eta \left(r\right)\right\}$ can only be realized by a so-called non-local point transformation (NLPT). Such a transformation, which incidentally is analogous to the one which realizes the so-called Einstein’s teleparallel approach to GR, is however a non-local transformation which is not applicable in the present context. As a consequence, Equation (16) effectively requires validity of the further variational constraint

(35) $$\delta H_{\mu \nu }\left(\Gamma \right)=0,$$

which in turn requires Equation (14) to hold identically in the whole spacetime $Q^{4}$, including (thanks to continuity) the boundary $\partial Q^{4}$. However, since in the original approach by Einstein the variations $\delta g_{\mu \nu }$ and $\delta \Gamma_{ik}^{l}$ are not considered as independent, as typically assumed instead in Palatini-type approaches, the issue arises of the actual validity of the constraint condition (14) in the framework of the functional setting ${\left\{g\right\}}_{C}$.

We notice that a similar possibility to the one proposed by Landau-Lifschitz is that advanced by Wald in Ref. [7], which concerns Equation (31) only and is based on the claim that the variation of the Ricci tensor should give rise to a divergence term acting on the variation $\delta g_{\mu \nu }$ which should therefore vanish by requiring that the same variation vanishes on the boundary. According to Ref. [7], “this term does not vanish for general variations where $g^{\mu \nu }$ is held fixed on the boundary, although it does vanish for variations where the first derivatives of $g^{\mu \nu }$ also are held fixed”.

*Choice B*—A further possibility concerned Equation (31) also advanced by Wald in Ref. [7], is based on the addition of an “ad hoc” extra surface-term contribution to the action functional (1), which is a function of $w^{\alpha }$ and is constructed in such a way to exactly balance the implicit contributions originating from the integration by parts performed in Equation (28). This approach however cannot be implemented to treat the constraint (32).

*Choice C*—Finally, another possible route, already anticipated above, arises in the context of the Palatini approach [13] provided the functional setting in the EH action functional (1) is properly prescribed. In such a case in fact, in contrast to the prescription (12) given above, the variations $\delta g_{\mu \nu }$ and $\delta \Gamma_{ik}^{l}$ are considered independent by construction (see for example [4] and the original derivation by Schroedinger [15]). Therefore in such a context the constraint condition (14) can in principle be legitimately set.

Regarding the possible choices indicated above there are however two crucial aspects to take into account. The first one is that, in all cases, the action principle based on the Einstein-Hilbert action may not be well posed [16,17]. In fact, since the Lagrangian is represented in terms of the variational Ricci scalar R(g(r)), derivation of the Einstein equations requires fixing both the metric tensor and its first derivative on the boundary, which is inconsistent with the same Einstein equations. The second one concerns Choice B. It refers to the fact that the prescription of boundary terms involves either assigning a suitable GR-frame (i.e., a coordinate setting, such as a foliatiation with respect to the coordinate time) for their representation in terms of variational variables, or to adopt a general representation in terms of connections $\Gamma_{bc}^{a}$ that have a non-tensorial character. Both possibilities necessarily violate manifest covariance. We stress that the same inconvenience is expected to arise in the case of alternative theories of GR (for example including torsion or f(R) effects) in which boundary terms are taken into account [18].

Based on the these consideration, apart possibly only the Palatini approach (which, however, adopting Frechet derivatives requires a suitable reformulation not reported here for brevity), the remaining tentative solutions *A* and *B* appear therefore questionable. The fundamental question which arises is therefore whether, in the context of the EH action principle (8) and subject to the boundary constraints (24), there is a possible way-out solution which effectively realizes an asynchronous variational principle for EFE and permits a consistent treatment of the whole constraint (28).

### 2.3. An Unconstrained Lagrangian Variational Principle

As we intend to prove here, the solution of the difficulties indicated above is represented by an unconstrained Lagrangian variational principle for EH theory. Inspired to a concept originally pointed out in Ref. [8], the new approach is represented by a different kind of variational approach which is based on the adoption of so-called “extremal” constraints. As explained below, such a kind of constraints are peculiar because they actually do not limit either the functional class of the variations δg(r) or that of the varied functions g(r), thus leaving both δg(r) and g(r) independent. As a consequence, the resulting variational principle remains, in a proper sense, unconstrained. As a further notable feature, contrary to the original EH setting, the issue of the well-posedness for the related variation principle does not arise here. In fact it is avoided alltogether thanks to the fact that, as explained in detailed below, in such a context the Ricci tensor is considered prescribed (i.e., it is not treated as variational).

Nevertheless, to reach the goal an extended functional setting, different from ${\left\{g\right\}}_{C}$, is required in which the variational symmetric tensor functions g(r) cannot generally be treated any more as metric tensors. As far as the functional setting is concerned, this implies that the covariant and countervariant components $g_{\mu \nu }$ and $g^{\mu \nu }$ are not required to satisfy the orthogonality condition (5), while a *background metric-tensor representation* is adopted. In other words, there must exist by construction a background tensor field, to be identified with a particular solution of EFE $\hat{g}\left(r\right)\equiv \left\{{\hat{g}}_{\mu \nu }\left(r\right)\right\}\equiv \left\{{\hat{g}}^{\mu \nu }\left(r\right)\right\}$ (see Equation (5)), which generates the geometric properties of the spacetime, in particular raising and lowering tensor indexes and prescribing the Christoffel symbols (and therefore the Ricci tensor). The functional setting is now identified with the unconstraint setting ${\left\{g\right\}}_{U}$ defined as

(36) $${\left\{g\right\}}_{U}\equiv \left\{\left.g\left(r\right)\equiv \hat{g}\left(r\right)+\delta g\left(r\right)\in C^{2}\left(Q^{4}\right)\right|g^{\mu \nu }={\hat{g}}^{\mu \alpha }{\hat{g}}^{\nu \beta }g_{\alpha \beta }\left(r\right),{\hat{g}}_{\mu \nu }{\hat{g}}^{\mu k}=\delta_{\nu }^{k}\right\},$$

where, in further difference with respect to ${\left\{g\right\}}_{C}$, all tensor indices are now raised and lowered by $\hat{g}\left(r\right)$.

The introduction of the setting (36) is required in order to satisfy the variational constraint condition. In fact, a necessary condition for the validity of the variational functional constraint condition (24) is to require that identically, that is, for arbitrary variations δg(r), the following two variational equations, that is, respectively

(37) $$\delta R_{\mu \nu }\equiv {\left.\frac{d}{d\alpha }R_{\mu \nu }\left(\hat{g}\left(r\right)+\alpha \delta g\left(r\right)\right)\right|}_{\alpha =0}=0,$$

and

(38) $$\delta R^{\mu \nu }\equiv {\left.\frac{d}{d\alpha }R^{\mu \nu }\left(\hat{g}\left(r\right)+\alpha \delta g\left(r\right)\right)\right|}_{\alpha =0}=0,$$

hold. In view of the definition of the Frechet derivative and ruling out possible constraint conditions on the variations δg(r), this means that the Ricci tensor, both in its covariant and countervariant components $R_{\mu \nu }$ and $R^{\mu \nu }$, *must necessarily be functions of* $\hat{g}\left(r\right)$ *only*. Therefore, in the unconstrained variational principle $R_{\mu \nu }$ should behave as an extremal 4-tensor in both its covariant and countervariant representations, that is,

(39) $$R_{\mu \nu }=R_{\mu \nu }\left(\hat{g}\left(r\right)\right)\equiv {\hat{R}}_{\mu \nu },$$

(40) $$R^{\mu \nu }=R^{\mu \nu }\left(\hat{g}\left(r\right)\right)\equiv {\hat{R}}^{\mu \nu },$$

with the background tensor field $\hat{g}\left(r\right)$ being identified with a particular solution of EFE (5), that is, *the extremal solution* $\hat{g}\left(r\right)$. Such a peculiar kind of constraints will be therefore referred to as extremal constraint conditions. However, one further notices that this requires also that

(41) $${\hat{R}}_{\mu \nu }={\hat{g}}_{\mu \alpha }\left(r\right){\hat{g}}_{\nu \beta }\left(r\right)R^{\alpha \beta }\left(\hat{g}\left(r\right)\right),$$

which means that the background tensor $\hat{g}\left(r\right)$ must be the metric tensor, that is, the tensor which raises and lowers tensor indices of all tensor fields. In fact, in order to preserve the 4-scalar property of the Lagrangian density (see Equation (3)), also the varied functions g(r) must behave accordingly (i.e., its indexes must be raised and lowered by $\hat{g}\left(r\right)$). This implies also that the orthogonality conditions (5) cannot generally be fulfilled because the simultaneous validity of Equations (39) and (40) is generally not permitted when the varied functions g(r) belong to (4). A fact which forces one to adopt the more general functional setting (36).

As a result, one concludes therefore that within the functional class ${\left\{g\right\}}_{U}$ necessarily Equations (37) and (38) are identically satisfied, without implying any constraint condition for the varied function g(r), nor on their variations δg(r). Then, in the same functional class, and based on the setting (39) and (40), the EH action integral (1) becomes

(42) $$\begin{array}{ccc} & & \left.S_{EH}^{\left(U\right)}\left(g,\hat{g}\right)\equiv \int_{Q^{4}}d^{4}rL_{var}\left(g,\hat{g}\right),\right.\\ & & \left.L_{var}\left(g,\hat{g}\right)=\sqrt{-\left|g\right|}\left[L_{EH}\left(g,\hat{g}\right)+L_{F}\left(g,\hat{g}\right)\right]\right., \end{array}$$

where following Equation (3) and including explicitly the dependence in terms of the extremal tensor field $\hat{g}\left(r\right)$, the integral $S_{EH}^{\left(U\right)}\left(g,\hat{g}\right)$ and

(43) $$L_{EH}\left(g,\hat{g}\right)=g^{\mu \nu }{\hat{R}}_{\mu \nu }-2\Lambda$$

identify respectively the unconstrained EH variational functional and the unconstrained variational Lagrangian density. The EH variational principle (8) thus reduces to the unconstrained variational principle

(44) $$\delta S_{EH}^{\left(U\right)}\left(g,\hat{g}\right)={\left.\frac{d}{d\alpha }S_{EH}^{\left(U\right)}\left(\hat{g}\left(r\right)+\alpha \delta g\left(r\right),\hat{g}\left(r\right)\right)\right|}_{\alpha =0}=0,$$

where the corresponding Euler-Lagrange equations become now

(45) $$\frac{\delta S_{EH}^{\left(U\right)}\left(g,\hat{g}\right)}{\delta g^{\mu \nu }}\equiv \frac{\partial L_{var}\left(g,\hat{g}\right)}{\partial g^{\mu \nu }}=0,$$

the partial derivative with respect to $g^{\mu \nu }$ being performed at constant $\hat{g}\equiv \left\{{\hat{g}}_{\mu \nu }\right\}$. Straightforward algebra then delivers the expected result, showing that the unconstrained Euler-Lagrange equations determined in this way indeed coincide identically with EFE (i.e., Equation (5)).

The result is striking because it shows that the constrained EH variational principle reduces in this case to an unconstrained one. This occurs because both the varied tensor functions g(r) and their variations δg(r) remain completely unaffected by the constraints (39) and (40), that is, provided g(r) belongs to a suitably chosen functional class ${\left\{g\right\}}_{U}$. The peculiarity arises because the same constraints are identically satisfied by themselves and do not affect the variational principle itself. It should be stressed that such a result has formal analogies with the Palatini variational principle because in both cases the Ricci tensor is regarded as independent of the variational tensor $g^{\mu \nu }$. The resemblance, however, stops here because, unlike the Palatini approach, here the connections are not considered variational.

### 2.4. An Equivalence Theorem

The equivalence of the two variational principles given above can readily be shown, provided they are both couched in the same functional setting, that is, ${\left\{g\right\}}_{U}$ and of course provided the boundary constraint condition (24) is also set (this is certainly not a restrictive condition considering that variational functions g(r) belonging to ${\left\{g\right\}}_{U}$ also obviously belong to ${\left\{g\right\}}_{C}$). In this regard an important preliminary remark must be made. This concerns the fact that the explicit variational contribution ${\left(\delta S_{EH}\right)}_{\mathrm{expl}}$ when evaluated with respect to the functional class ${\left\{g(r)\right\}}_{U}$ takes the same representation given by Equation (4) (the reason being that the identity (19) still holds). This makes possible to evaluate in the same functional class ${\left\{g\right\}}_{U}$ the difference functional

(46) $$\Delta S_{EH}\left(g,\hat{g}\right)\equiv S_{EH}\left(g\right)-S_{EH}^{\left(U\right)}\left(g,\hat{g}\right)=\int_{Q^{4}}d^{4}r\Delta L_{var}\left(g,\hat{g}\right),$$

(47) $$\Delta L_{var}\left(g,\hat{g}\right)\equiv L_{var}\left(g\right)-L_{var}\left(g,\hat{g}\right).$$

Thus, in view of Equations (4) and (44) it follows that the variational principle $\delta \Delta S_{EH}\left(g,\hat{g}\right)=0$ yields identically:

(48) $$\int_{Q^{4}}d^{4}r\delta \Delta L_{var}\left(g,\hat{g}\right)=0.$$

This means therefore that when setting $g\left(r\right)=\hat{g}\left(r\right)$ the previous equation must be satisfied identically. As a consequence, $\Delta L_{var}\left(g,\hat{g}\right)$ represents effectively a gauge contribution to the variational Lagrangian density.

The implication is therefore that the boundary-constrained EH variational principle Equation (8) and the unconstrained one (44) are completely equivalent, both yielding the same form of EFE. This means also that the constraint conditions (37) and (38) should be interpreted as *natural constraint conditions*, in the sense that they are necessarily satisfied in the framework of EH theory. In other words, it is always possible to get rid altogether of the variations $\delta R_{\mu \nu }$ which enter the Euler-Lagrange equations. This provides effectively a new perspective in EH theory, since the property holds for arbitrary coordinates systems and is therefore frame-independent.

### 2.5. Covariance and the Issue of Missing Gauge Invariance

To complete the analysis, the property of covariance and the issue of (missing) gauge invariance of the EH theory is briefly addressed here. First, the manifest covariance and covariance properties of the EH variational principle, Lagrangian density and of the corresponding EFE, are readily established. These properties follow from two basic ingredients:

(1) The 4-tensor nature of the EH action functional, either in its constrained or unconstrained forms, that is, respectively given by Equations (8) and (44).

(2) The fact that variations are performed with respect to a tensor field, that is, $g_{\mu \nu }$.

Thanks to the 4-scalar nature of the (invariant) volume element dΩ (see Equation (2)) and similarly thanks to the assumption that the Langrangian densities are by construction 4-scalars, either if represented in terms of their constrained or unconstrained forms, namely $L_{EH}\left(g\right)+L_{F}\left(g\right)$ and $L_{EH}\left(g,\hat{g}\right)+L_{F}\left(g,\hat{g}\right)$ respectively, implies that necessarily the EH variational principle (i.e., the action integral) and EFE are consistent with the principle of manifest covariance. As an implication, the covariant nature of the theory is established at once. In fact, given an arbitrary local point transformation, that is, an arbitrary diffeomorphism

(49) $$r\to r^{\prime }=r^{\prime }\left(r\right)$$

which maps the spacetime $\left\{Q^{4},\hat{g}\left(r\right)\right\}$ in itself, both the EH variational principle and the corresponding EFE (both written equivalently either in unconstrained or constrained forms) are covariant.

A different consideration however holds for the Lagrangian density, which represents the effective quantity that generates the EL equations after taking variations and which, for this reason, should encode the physical properties of the field. In the case of EH variational principle the variational Lagrangian density $L_{var}\left(g\right)$ defined by Equation (16) is not a 4-scalar, since the contribution of the determinant $\sqrt{-\left|g\right|}$ is not either. This feature is unusual in field theory, because it means that the resulting EL equations, namely EFE, are determined partly by a contribution carried by the Lagrangian function $L_{EH}\left(g\right)$ and partly by a non-tensor contribution which defines the differential volume element of the action integral and which is independent of the actual realization of the same Lagrangian function. This is not a marginal aspect, but rather implies serious consequences on the theoretical framework of variational theory for GR. In particular:

(1) Either the constrained or unconstrained symbolic Euler-Lagrange equations, namely (17) and (45), are not set in manifest-covariant form. This feature notwithstanding, however, upon evaluating explicitly the functional derivatives in the two cases one immediately realizes that the corresponding (and equivalent) EFE represented by Equation (5) are manifestly covariant too, that is, are set in 4-tensor form.

(2) The EH asynchronous variational formulation lacks a basic gauge invariance property which should however characterize standard variational theories of continuum classical fields. We refer here to the gauge-invariance property provided by the trivial gauge transformation acting on the variational field Lagrangian *L* in terms of an arbitrary constant 4-scalar C=const. by means of the transformation

(50) L→L+C.

In the standard theory of variational principles the addition of a constant in the Lagrangian must leave invariant the extremal equations. In contrast, in the context of asynchronous EH principle it follows that, when Equation (50) applies, any Lagrangian density L of the type $L=\sqrt{-\left|g\right|}L$ transforms necessarily as

(51) $$L\to L+\sqrt{-\left|g\right|}C.$$

Therefore, the introduction of the additive constant *C*, instead of leaving invariant the Euler-Lagrange equations, changes the functional form of EFE, thus generating a non-vanishing contribution in the same extremal equations which is equal to $-\frac{1}{2}Cg_{\mu \nu }$. However, the ensuing lack of such gauge-invariance property - which incidentally affects both the EH and ADM theories—is not simply a mere unfortunate inconvenience. In fact it violates a fundamental requirement usually regarded as a cornerstone of continuum classical and quantum field theory. We stress once more that this feature is ultimately related to the adoption of a non-tensorial variational Lagrangian density, which depends on $\sqrt{-\left|g\right|}$ associated with the 4-volume differential element, and gives rise to a continuum field theory which is intrinsically non-gauge invariant. However, the property of gauge invariance should be regarded as a mandatory feature of variational field theories in general, to be fulfilled both by variational and extremal continuum fields. As a consequence, also the variational functional and the corresponding variational Lagrangian, together with the corresponding extremal quantities, should be necessarily determined up to a suitable gauge contribution. The violation of this property in the asynchronous EH variational theory is therefore a conceptual and theoretical obstacle to the realization of a truly manifestly-covariant variational formulation of EFE.

## 3. The ADM Variational Approach

Let us now briefly recall the well-known ADM theory, which in mainstream literature is commonly credited for realizing a viable Hamiltonian formulation for GR. Formulated in 1959 [3,4] and heavily inspired by previous work by Dirac [19,20], it partly relies on some of his mathematical formalism, especially Dirac’s customary identification of canonical momenta in terms of coordinate-time partial derivatives and Dirac’s theory of constrained dynamics [21].

For the purposes of the present investigation (and the economy of presentation) it is sufficient to consider here only the so-called “Hamiltonian realization” of ADM theory. The variational approach, whose starting point is based on a particular coordinate representation of the EH Lagrangian variational principle, is based on the following steps:

- The prescription of the variational functional, which is identified with the EH action functional (1).
- The prescription of the functional class ${\left\{g(r)\right\}}_{C}$ in which the varied functions take the generic form $g\left(r\right)\equiv g_{extr}\left(r\right)+\delta g\left(r\right)$, where $g_{extr}\left(r\right)$ is identified with a suitable extremal tensor field.
- The prescription of ADM variables in terms of the varied tensor function g(r) (and hence of the extremal tensor field $g_{extr}\left(r\right)$ too).
- The representation of the EH Lagrangian density in ADM variables.

In detail, once having prescribed the variational functional, the second step is based on the replacement of the variational tensor $g_{\mu \nu }\left(r\right)$, required to belong to ${\left\{g(r)\right\}}_{C}$, with a suitable set of non-4-tensor variational variables, identified with the set $\left(h_{ab}\left(r\right),N\left(r\right),N_{a}\left(r\right)\right)$ to be denoted as *ADM Lagrangian variables*, where $h_{ab}$ is the 3×3 variational matrix (the Latin indices a,b run from 1 to 3, while the greek ones range as usual from 0 to 3), while *N* and $N^{a}$ are respectively the *lapse* function and the *shift* 3-vector. More precisely, the ADM variables are defined by suitably prescribing the set of coordinates (GR-frame) $r\equiv \left\{r^{\mu }=\left(ct,r\right)\right\}$ and the components of the variational tensor $g_{\mu \nu }\left(r\right)$ as follows:

(52) $$\begin{array}{ccc} g_{00}\left(r\right) & = & \frac{1}{c^{2}}\left(-N^{2}+N_{a}N^{a}\right),\\ g_{0a}\left(r\right) & = & g_{a0}=\frac{1}{c}N_{a},\\ g_{ab}\left(r\right) & = & h_{ab}. \end{array}$$

Notice that a priori all components of *g*, as well of $\left(h_{ab}\left(r\right),N\left(r\right),N_{a}\left(r\right)\right)$ are considered functions of the 4-position $r=\left\{r^{\mu }\right\}$, that is, generally dependent on both time and spatial coordinates. Then, direct calculation shows that in whole generality the identity

(53) $$\sqrt{-\left|g\right|}⟶N\left(r\right)\sqrt{\left|h(r)\right|}$$

must hold.

The fundamental aspect to highlight here is that, in view of the outcomes of Section 2, there can exist actually two different admissible representations of the Lagrangian density in ADM variables, which are related to the two possible choices of the EH Lagrangian densities $L_{var}\left(g_{\mu \nu }\right)$ and $L_{var}\left(g_{\mu \nu },{\hat{g}}_{\mu \nu }\right)$ (see Equations (16) and (42) respectively) corresponding to the boundary-constrained and unconstrained Lagrangian EH variational principles stated above. In the following, in order to question the nature of the ADM Hamiltonian theory for EFE, we shall therefore consider separately the two settings.

### 3.1. The Constrained ADM Variational Formulation

Let us consider first the relationship in terms of $L_{var}\left(g_{\mu \nu }\right)$ originally established by Arnowitt, Deser and Misner (ADM theory [4]). Such an approach will be hereon referred to as *constrained ADM variational formulation*. The task involves suitably representing in terms of ADM variables both $S_{EH}\left(g\right)$ and the corresponding variational Lagrangian density $L_{var}$ (see Equation (16) above), assuming the functional setting ${\left\{g(r)\right\}}_{C}$. To this end the 4-dimensional integral is split as a time integral (by setting also c=1) and a surface integral on the (constant-time) 3-surface section $\Sigma_{t}$, where *t* is the coordinate-time, by representing $d^{4}r$ as $d^{4}r=dtd\Sigma$, with dΣ being the surface element on $\Sigma_{t}$. For simplicity, we consider here the vacuum case with non-vanishing cosmological constant (Λ≠0).

Starting point is the prescription of the ADM variational functional, namely

(54) $$S_{ADM}\left(G\right)=\int dt\int_{\Sigma_{t}}d\Sigma L_{ADM}\left(G\right),$$

and the related ADM variational Lagrangian density $L_{ADM}\left(G\right)$, which are identified according to

(55) $$\left\{\begin{array}{c} S_{ADM}\left(G\right)\equiv S_{EH}\left(g\right),\\ L_{ADM}\left(G\right)=L_{var}\left(g\right). \end{array}\right.$$

Here, G(r) denotes the set of ADM canonical variables $G\left(r\right)\equiv \left\{h_{ab},p^{ab},N,N_{a}\right\}$, with $\left\{h_{ab},N,N_{a}\right\}$ being the Lagrangian variables determined by inversion of Equation (52). Then, if it is assumed that g(r) belongs to the functional setting ${\left\{g\right\}}_{C}$ (defined by Equation (4)), this means that the corresponding functional setting $\left\{G(r)\right\}$ should be prescribed accordingly.

Notice that here the ADM variational Lagrangian density $L_{ADM}\left(G\right)$ must be identified with the EH Lagrangian density $L_{var}\left(g_{\mu \nu }\right)$ (see Equation (16)). This can be shown to require

(56) $$L_{ADM}\left(G\right)\equiv p^{ab}{\overset{\cdot }{h}}_{ab}-NH_{⊥}\left(G\right)-N_{a}H^{a}\left(G\right),$$

where the notations are standard. Thus, in particular, $p^{ab}$ are suitably-defined “canonical momenta” conjugate to $h_{ab}$, ${\overset{\cdot }{h}}_{ab}$ is the Lie derivative with respect to time of $h_{ab}$, which according to the choice of ADM variables in such a reference frame coincides with the partial time derivative ${\overset{\cdot }{h}}_{ab}\equiv \frac{\partial }{\partial t}h_{ab}$, while $H_{⊥}$ and $H^{a}$ are the so-called “Hamiltonians”

(57) $$H_{⊥}=\sqrt{\left|h\right|}\left[{-}^{\left(3\right)}R-2\Lambda +{\left|h\right|}^{-1}p^{ab}p_{ab}-\frac{1}{2}{\left|h\right|}^{-1}p^{2}\right],$$

(58) $$H^{a}=-2\sqrt{\left|h\right|}D_{b}\left({\left|h\right|}^{-1/2}p^{ab}\right),$$

where $p=p_{a}^{a}$ and $D_{c}$ identifies the covariant derivative prescribed on the section $\Sigma_{t}$, which when acting on $p_{b}^{a}$ is defined as $D_{c}p_{b}^{a}=h_{d}^{a}h_{b}^{e}h_{c}^{f}\nabla_{f}p_{e}^{d}$. Furthermore, it is important to notice that ${}^{\left(3\right)}R$ represents the spatial curvature scalar and is defined as ${}^{\left(3\right)}R=h^{lm(3)}R_{lm}$, where ${}^{\left(3\right)}R_{lm}$ is the 3-dimensional Ricci tensor, which is defined in terms of the corresponding 3-dimensional Riemann tensor as ${}^{\left(3\right)}R_{lm}{=}^{\left(3\right)}R_{lkm}^{k}$. As such, ${}^{\left(3\right)}R_{lm}$ still depends on first and second-order partial spatial derivatives of the Lagrangian ADM fields, namely it is generally of the form

(59) $${}^{\left(3\right)}R_{lm}{=}^{\left(3\right)}R_{lm}\left(h_{ab},\partial_{i}h_{ab},\partial_{i}\partial_{j}h_{ab}\right).$$

More precisely, it is defined as

(60) $${}^{\left(3\right)}R_{lm}{=}^{\left(3\right)}R_{lkm}^{k}=\partial_{k}\gamma_{lm}^{k}-\partial_{m}\gamma_{kl}^{k}+\gamma_{kn}^{k}\gamma_{lm}^{n}-\gamma_{mn}^{k}\gamma_{kl}^{n},$$

with $\gamma_{jk}^{i}$ denoting the 3-dimensional Christoffel symbols

(61) $$\gamma_{jk}^{i}=\frac{1}{2}h^{il}\left(\partial_{j}h_{kl}+\partial_{k}h_{jl}-\partial_{l}h_{jk}\right).$$

Finally, let us introduce the parametrized varied functions

(62) $${\left(G\right)}_{extr}+\alpha \delta G\equiv \left(h_{ab}\left(r,\alpha \right),p^{ab}\left(r,\alpha \right),N\left(r,\alpha \right),N_{a}\left(r,\alpha \right)\right),$$

where α is a real parameter $\alpha \in \left[-1,1\right]$ and

(63) $$\left\{\begin{array}{c} h_{ab}\left(r,\alpha \right)={\left(h_{ab}\right)}_{extr}+\alpha \delta h_{ab},\\ p^{ab}\left(r,\alpha \right)={\left(p^{ab}\right)}_{extr}+\alpha \delta p^{ab},\\ N\left(r,\alpha \right)={\left(N\right)}_{extr}+\alpha \delta N,\\ N_{a}\left(r,\alpha \right)={\left(N_{a}\right)}_{extr}+\alpha \delta N_{a}, \end{array}\right.$$

where $\delta h_{ab}\equiv \delta g_{ab},$ while ${\left(G\right)}_{extr}\equiv \left({\left(h_{ab}\right)}_{extr},{\left(p^{ab}\right)}_{extr},{\left(N\right)}_{extr},{\left(N_{a}\right)}_{extr}\right)$ denotes the extremal solution (see below) which extremizes the variational functional. Thus, the ADM variational principle in terms of the Frechet derivative becomes:

(64) $$\delta S_{ADM}\left(G\right)\equiv {\left.\frac{d}{d\alpha }S_{ADM}\left({\left(G\right)}_{extr}+\alpha \delta G\right)\right|}_{\alpha =0}=0,$$

which must hold for arbitrary (linearly) independent variations $\delta h_{ab}$, $\delta p^{ab}$, δN and $\delta N_{a}$ defined according to the functional setting. About the notations, we stress that the extremal tensor fields ${\left(h_{ab}\right)}_{extr}$, ${\left(p^{ab}\right)}_{extr}$, ${\left(N\right)}_{extr}$ and ${\left(N_{a}\right)}_{extr}$ are considered solutions of the corresponding Euler-Lagrange equations (see below Equation (65)). The same fields, based on Equation (52) determine also the corresponding extremal tensor field $g_{extr}\left(r\right)$ which in principle needs not to coincide with $\hat{g}\left(r\right)$.

The formal derivation of the Euler-Lagrange equations determined by (64) is reported in detail by several authors (see for example Wald [7]). According to these references this yields for the extremal ADM fields $G={\left(G\right)}_{extr}$ the set of *ADM initial-value equations* which is realized respectively by

(65) $$\left\{\begin{array}{c} {\overset{\cdot }{h}}_{ab}-N\frac{\partial }{\partial p^{ab}}H_{⊥}-N_{c}\frac{\partial }{\partial p^{ab}}H^{c}=0,\\ -{\overset{\cdot }{p}}^{ab}-N\frac{\partial }{\partial h_{ab}}H_{⊥}-N_{c}\frac{\partial }{\partial h_{ab}}H^{c}=0,\\ H_{⊥}=0,\\ H^{a}=0, \end{array}\right.$$

and by the initial conditions

(66) $$(h_{ab}\left(t_{o}\right),p^{ab}\left(t_{o}\right)),$$

with $t_{o}$ denoting a prescribed (but arbitrary) initial coordinate time. Thus, in particular, the first two equations in (65) represent two Hamilton-like evolution equations for the conjugate variables $\left(h_{ab},p^{ab}\right)$, formality representing Lagrangian generalized coordinates and conjugate momenta. The remaining two equations are instead respectively a 3-scalar and a 3-vector constraint equations expressed in terms of so-called “Hamiltonian” functions $H_{⊥}$ and $H^{a}$, which are required to vanish identically for the extremal values of the ADM variables $\left(h_{ab},p^{ab},N,N_{a}\right)$. The basic implication which follows is therefore that, from the ADM variational principle (64), the extremal ADM variables ${\left(G\right)}_{extr}$, in particular $\left({\left(h_{ab}\right)}_{extr},{\left(p^{ab}\right)}_{extr}\right)$, are not independent being subject to the previous constraint equations (last two equations in Equation (65)).

We notice that in the literature (see [4,7]) it is claimed that in the case of vacuum the same ADM initial value equations “are equivalent to the vacuum Einstein equation”. However, it is important to recognize that the initial conditions (66) are in principle free. On the other hand, one can show that the Lagrange multipliers $N,N_{a}$ are uniquely determined by requiring that initially (at $t_{o}$) they satisfy the said constraint equations [4]. As a consequence, as shown below, the solution of the ADM Euler-Lagrange Equations (65) and (66) is necessarily non unique. The obvious but nevertheless still nontrivial consequence is that the same system of equation does not necessarily coincide identically with EFE, unless a particular choice of initial conditions (66), to be therefore appropriately determined, is actually selected.

The other important remark concerns the customary literature identification of the ADM initial-value problem (65) and (66) with a Hamiltonian system in the proper sense, to which therefore the customary formalism of Hamilton and Hamilton-Jacobi theories should apply. For such an identification to be possible, however, the same ADM initial-value problem *should identify a set of first order, evolution ODEs with respect to the ADM variables*$G\equiv \left\{h_{ab},p^{ab},N,N_{a}\right\}$.

However, such a statement is manifestly incorrect. The reason is as follows. According to the standard notion of Hamiltonian system, the Hamilton equations should depend only on the generalized coordinates and the conjugate momenta, and not contain additional (partial) derivatives of the coordinates. Nevertheless, the ADM Euler-Lagrange equations, just as the original EFE (recalled above by Equation (5)), still contain second-order partial derivatives acting on the Lagrangian ADM variables $h_{ab}$. The crucial term which carries such a kind of contributions is the spatial curvature ${}^{\left(3\right)}R_{ab}$ (in EFE it is the Ricci tensor), which in fact as shown by Equation (59) (see also definition given by Equation (60)) depends on first and second order partial spatial derivatives of $h_{ab}$.

Thus, first one notices that in the ADM Euler-Lagrange Equation (65) ${}^{\left(3\right)}R_{ij}$ depends on $h_{ab}$ too, so that

(67) $${\frac{\partial }{\partial h_{ij}}}^{\left(3\right)}R\neq^{\left(3\right)}R_{ij},$$

contrary to what assumed in the standard ADM approach. Furthermore, there are two equations, namely respectively the second and the third ones, which identify PDEs instead of ordinary differential equations as required for their identification in terms of a Hamiltonian system.

Incidentally, however, there is a further problematic (and dubious) aspect of the ADM approach to be mentioned.

This concerns the issue of the variational treatment of the same 3-tensor represented either in covariant or countervariant representations, that is, ${}^{\left(3\right)}R_{lm}$ and ${}^{\left(3\right)}R^{lm}$. In fact, as indicated above in Equation (59), both representations of the same 3-tensor contains explicit dependencies in terms of first and second-order partial derivatives of $h_{ab}$ with respect to the spatial coordinates. In fact, for consistency with the functional setting established above, the variation under the integral (54) should be again performed in terms of the Frechet derivative. This includes the variation of the 3-tensor ${}^{\left(3\right)}R_{lm}$ (or ${}^{\left(3\right)}R^{lm}$). Notice in fact that the same 3-tensor cannot be regarded as constant when it is considered under the integral (see also related discussion in case of *Choice A* in Section 2.2 above). Furthermore, in view of the previous definition (see Equation (60)), the functional variation of $\delta^{\left(3\right)}R_{lm}$ (similarly for ${}^{\left(3\right)}R^{lm}$) necessarily takes the form:

(68) $$\begin{array}{ccc} \delta^{\left(3\right)}R_{lm} & = & {}^{\left(3\right)}R_{lm}\left(\delta g_{ab},\partial_{i}{\left(h_{ab}\right)}_{extr},\partial_{i}\partial_{j}{\left(h_{ab}\right)}_{extr}\right)\\ & & \left.{+}^{\left(3\right)}R_{lm}\left({\left(h_{ab}\right)}_{extr},\partial_{i}\delta g_{ab},\partial_{i}\partial_{j}{\left(h_{ab}\right)}_{extr}\right)\right.\\ & & {+}^{\left(3\right)}R_{lm}\left({\left(h_{ab}\right)}_{extr},\partial_{i}{\left(h_{ab}\right)}_{extr},\partial_{i}\partial_{j}\delta g_{ab}\right), \end{array}$$

which therefore depends, besides $\delta g_{ab}$, also on the first and second-order (spatial) partial derivatives $\partial_{i}\delta g_{ab}$ and $\partial_{i}\partial_{j}\delta g_{ab}$. On the other hand, for the validity of the variational principle (64) the dependences in terms of the first and second partial derivatives should not appear. Instead, for the validity of the Euler-Lagrange equations themselves reported above (i.e., Equation (65)) all contributions should actually be considered ignorable, namely requiring instead

(69) $$\delta^{\left(3\right)}R_{ab}=0,$$

(70) $$\delta^{\left(3\right)}R^{ab}=0.$$

In both cases this implies that they should satisfy *extremal constraint conditions* of the type

(71) $${}^{\left(3\right)}R_{ab}={}^{\left(3\right)}R_{ab}\left({\left(h_{ab}\right)}_{extr},\partial_{i}{\left(h_{ab}\right)}_{extr},\partial_{i}\partial_{j}{\left(h_{ab}\right)}_{extr}\right),$$

(72) $${}^{\left(3\right)}R^{ab}={}^{\left(3\right)}R^{ab}\left({\left(h_{ab}\right)}_{extr},\partial_{i}{\left(h_{ab}\right)}_{extr},\partial_{i}\partial_{j}{\left(h_{ab}\right)}_{extr}\right),$$

with ${\left(h_{ab}\right)}_{extr}$ denoting the extremal tensor field $h_{ab}\left(r\right)$. Nevertheless, since $R^{ab}$ and $R_{ab}$ are related by assumption via the 3-tensor $h_{ab}\left(r\right)$ ($h^{ab}\left(r\right)$) which lowers (raises) their tensor indices, it is obvious that a requirement cannot be consistent with the functional setting of the ADM approach indicated above (4) (see also the analogous discussion in Section 2.3). The alternative, that is, which occurs when these dependencies are retained, has the *so-to-say*“*unpleasant*” side-effect of simply destroying the validity of the ADM variational principle (64). Indeed, $\delta^{\left(3\right)}R_{ab}$ gives rise in such a case (through the Frechet derivative $\delta^{\left(3\right)}R_{ab}$) to the appearance of first and second-order partial derivatives of the variations $\delta h_{ab}$.

In conclusion, the suggested implication is that the ADM initial value equations are formally correct only provided the variational contribution of $\delta^{\left(3\right)}R_{ab}$ and $\delta^{\left(3\right)}R^{ab}$ are both set to zero (which amounts to introduce a hidden constraint), while—in addition—the contribution of the extremal term ${}^{\left(3\right)}R_{ab}\left({\left(h_{ab}\right)}_{extr}\right)$ gives anyway rise (as in the case of EFE) to the appearance of second-order partial derivatives of ${\left(h_{ab}\right)}_{extr}$, thus yielding an intrinsically non-Hamiltonian PDE.

We stress that such features cannot be corrected by adopting a perturbation theory, that is, in which the spatial curvature is treated to leading order as constant. In fact perturbative schemes of this type do not converge everywhere (for example near singularities) and therefore do not appear reliable for the establishment of a quantum theory of gravitation based on the ADM formulation of GR.

### 3.2. An Unconstrained Lagrangian Variational Formulation for the ADM Representation

As anticipated, we now consider the relationship in terms of the unconstrained Lagrangian EH variational principle which is characterized by the corresponding Lagrangian density $L_{var}\left(g_{\mu \nu }\right)$ (see Equation (42)). As we intend to show this provides an alternate route for the prescription of the ADM variables and at the same time a new possible realization of ADM theory.

This is based on the unconstrained formulation of EH theory pointed out above (see Section 2.2) and on the construction of the variational functional $S_{EH}\left(g,\hat{g}\right)$ (see Equation (42)). The new approach consists now in representing $S_{EH}\left(g,\hat{g}\right)$ and the corresponding variational Lagrangian density $L_{var}\left(g,\hat{g}\right)$ in terms of ADM variables, thus prescribing the new ADM functional and Lagrangian density according to

(73) $$\left\{\begin{array}{c} S_{ADM}\left(G,\hat{g}\right)\equiv S_{EH}\left(g,\hat{g}\right),\\ L_{ADM}\left(G,\hat{g}\right)\equiv L_{var}\left(g,\hat{g}\right). \end{array}\right.$$

Here the action functional and variational Lagrangian density (respectively $S_{EH}\left(g,\hat{g}\right)$ and $L_{var}\left(g,\hat{g}\right)$) are now both defined with respect to the functional class ${\left\{g\left(r\right)\right\}}_{U}$. This feature permits us to fulfill the variational constraint (24), according to which the Ricci tensor is considered extremal, that is, it is set equal to ${\hat{R}}^{\mu \nu }\equiv R^{\mu \nu }\left(\hat{g}\left(r\right)\right)$. Thus, upon invoking the unconstrained EH variational principle (44), this implies identically

(74) $$\delta S_{ADM}\left(G,\hat{g}\right)=0.$$

For clarity and to distinguish it from the standard ADM variational principle indicated above (i.e., Equation (64)), this will be referred to here as the unconstrained ADM Lagrangian variational principle. If one considers the case of vacuum endowed with a non-vanishing cosmological constant, then it follows in particular

(75) $$L_{ADM}\left(G,\hat{g}\right)=N\sqrt{\left|h\right|}L_{EH}\left(g,\hat{g}\right),$$

where by construction $g^{\mu \nu }{\hat{R}}_{\mu \nu }=g_{\mu \nu }{\hat{R}}^{\mu \nu }$, so that $L_{EH}\left(g,\hat{g}\right)$ is given by Equation (43). Hence, in terms of the ADM representation (52) it follows:

(76) $$g_{\mu \nu }{\hat{R}}^{\mu \nu }=\frac{1}{c^{2}}\left(-N^{2}+N_{a}N^{a}\right){\hat{R}}^{00}+\frac{2}{c}N_{i}{\hat{R}}^{0i}+h_{ab}{\hat{R}}^{ab}.$$

To determine the corresponding Euler-Lagrange equations we stress that, unlike the variational principle (64), the calculations must now be performed with respect to the functional class ${\left\{g(r)\right\}}_{U}$ so that they exactly coincide with EFE (i.e., Equation (5) upon setting $T_{\mu \nu }=0$), thus yielding

(77) $$R_{\mu \nu }-\left(\frac{1}{2}R-\Lambda \right)g_{\mu \nu }=0,$$

where by construction $R_{\mu \nu }={\hat{R}}_{\mu \nu }$, $R=\hat{R}$ and $g_{\mu \nu }={\hat{g}}_{\mu \nu }$. The required Euler-Lagrange equations for the extremal ADM fields are then simply:

(78) $$\begin{array}{ccc} {\hat{R}}_{00}-\left(\frac{1}{2}\hat{R}-\Lambda \right)\frac{1}{c^{2}}\left(-N^{2}+N_{a}N^{a}\right) & = & 0,\\ {\hat{R}}_{0a}-\left(\frac{1}{2}R-\Lambda \right)\frac{1}{c}N_{a} & = & 0,\\ {\hat{R}}_{ab}-\left(\frac{1}{2}\hat{R}-\Lambda \right)h_{ab} & = & 0, \end{array}$$

which therefore depend only on the Lagrangian ADM fields $D\equiv (h_{ab},N,N_{a})$. Here it is understood that the representation of the extremal Ricci tensor must be expressed as well in terms of ADM variables through the customary definition of Christoffel symbols in terms of the metric tensor. We notice here that:

- The Euler-Lagrange Equation (78) coincide with EFE (see Equation (5)) when expressed in terms of the Lagrangian ADM fields $D\equiv (h_{ab},N,N_{a})$.
- The extremal solution $D_{extr}\equiv {\left(h_{ab},N,N_{a}\right)}_{extr}$ coincides necessarily with $D_{extr}\equiv \left({\hat{h}}_{ab},\hat{N},{\hat{N}}_{a}\right)$, where ${\hat{h}}_{ab},\hat{N}$ and ${\hat{N}}_{a}$ denote the ADM fields expressed in terms of $\hat{g}\left(r\right)$, that is, the background metric tensor and the solution of EFE. Therefore, by inversion of Equation (52), their representation in terms of $\hat{g}\left(r\right)$ is necessarily provided by
  (79) $$\begin{array}{ccc} \hat{N}\left(r\right) & = & c\sqrt{{\hat{g}}_{0a}\left(r\right){\hat{g}}^{0a}\left(r\right)-{\hat{g}}_{00}\left(r\right)},\\ {\hat{N}}_{a}\left(r\right) & = & c{\hat{g}}_{0a}\left(r\right)=c{\hat{g}}_{a0}\left(r\right),\\ {\hat{h}}_{ab}\left(r\right) & = & {\hat{g}}_{ab}\left(r\right). \end{array}$$
- Since the extremal Ricci tensor ${\hat{R}}_{\mu \nu }$ depends on second-order partial derivatives of $\hat{g}\left(r\right)$, Equation (78) must be intended again, in analogy to EFE, as implicit PDEs for the extremal Lagrangian ADM fields $D_{extr}\equiv \left({\hat{h}}_{ab},\hat{N},{\hat{N}}_{a}\right)$.

Let us now address the issue of the relationship between EFE and the same ADM Euler-Lagrange equations. This is done by requiring the simultaneous validity of the two ADM variational approaches indicated above and realized respectively according to the constrained and unconstrained Lagrangian variational principles. First, we notice that Equation (79) necessarily provides a particular solution of Equations (65) and (66) in which, however, $p^{ab}$ remains apparently undetermined. However, one can show that the second equation in (65) yields a differential equation for $p^{ab}$ subject to a suitable initial condition for $p^{ab}\left(t_{o}\right)$. In particular, in the case $\hat{g}\left(r\right)$ is stationary, by setting $p^{ab}\left(t_{o}\right)=0$ it follows that $p^{ab}$ remains everywhere identically zero and therefore is uniquely determined too.

On the contrary, if different initial conditions (66) are selected then it is obvious that generally the solution of the ADM initial value Equations (65) and (66) may not coincide with $\hat{g}\left(r\right)$. The proof is immediate. In fact this manifestly occurs if, in contrast with the third equation in (78), the initial condition for $h_{ab}\left(t,x\right)$, namely $h_{ab}\left(t_{o},x\right)$, is chosen to be

(80) $$h_{ab}\left(t_{o},x\right)\neq {\hat{g}}_{ab}\left(t_{o},x\right).$$

As a consequence it is concluded that the same ADM initial value equations and EFE (see Equation (5)) do not generally coincide. This conclusion is important for two main reasons. The first one is that it rules out a possible misunderstanding (see also Wald’s citation in the previous section) according to which vacuum EFE and the ADM initial value equations should coincide always, that is, independent of the initial conditions. The second one is because it permits us to address the problem of the role of the variational constraints (i.e., Equations (6), (39) and (40)) in ADM theory. In fact, the previous conclusion shows that the same ADM initial value equations do not generally satisfy the variational constraints. However, this happens if proper initial conditions are chosen for the ADM fields.

Finally, despite being covariant with respect to the group of local point transformations (LPT), it should be added that ADM theory is not manifestly-covariant with respect to the same group. As a consequence, it is not objective, so that the relevant equations, and in particular the coordinate time, are generally not preserved when introducing a generic LPT. In other words, the ADM variational variables, either Hamiltonian or Lagrangian ones, do not have a tensorial character. This is similar to what occurs in the case of the Palatini variational approach to GR, where variational variables include the connections, which are not 4-tensors. Nevertheless, in difference with the Palatini approach where the variations of the connections are by themselves 4-tensors together with the resulting Euler-Lagrange equations, the variations of ADM variables and the corresponding Euler-Lagrange equations (i.e., respectively either (65) or (78)) do not have a tensorial character either.

## 4. Synchronous and Manifestly-Covariant Hamiltonian Theory of GR

Given validity of the outcomes pointed out in previous sections, here an alternative to the non-manifestly covariant, non-gauge invariant and non-Hamiltonian ADM theory is provided. In this regard, it is appropriate to recall what is actually its starting point. This is given by the common belief that is epitomized for example by the original sentence due to Wald [7] according to which: “*... a Hamiltonian formulation of a field theory requires a breakup of spacetime into space and time. Indeed the first step...consists of choosing a time function t and a vector field* $t^{\alpha }$ *(the “flow of time”) on a spacetime such that the (space) surfaces* $\Sigma_{t}$ *of constant t are spacelike Cauchy surfaces (and) such that* $t^{\alpha }\nabla_{\alpha }1=1$.” The underlying philosophy of the whole ADM approach and the same sentence are, however, fundamentally incorrect. First, because in field theory the notion of manifest covariance, and more precisely that of manifestly-covariant Hamiltonian approach, are already well known, being due to deDonder and Weyl [22,23,24,25,26,27,28]. Second because, already in the context of classical and quantum mechanics, the adoption of proper time in place of the coordinate time *t* is an obvious matter-of-fact method which affords the realization of manifesly-covariant representations of relativistic Hamiltonian systems.

However, manifest covariance requires the prescription of a background spacetime viewpoint by means of a suitable functional setting on the class of varied functions g(r). In fact, in order to establish the very notion of manifest covariance it is obviously necessary to specify in advance the spacetime with respect to which the same property holds. Thus, identifying such a spacetime in terms of a differential manifold $\left\{Q^{4},\hat{g}\left(r\right)\right\}$, the task involves to suitably prescribe the corresponding background metric tensor $\hat{g}\left(r\right)$ with respect to some prescribed GR-frame $r\equiv \left\{r^{\mu }\right\}$. For definiteness, here the spacetime will be intended as a 4-dimensional time-oriented Riemannian differential manifold. Accordingly, $\hat{g}\left(r\right)$ determines the geometric properties of spacetime so that, besides raising and lowering tensor indices, it must prescribe also the covariant derivatives and the Christoffel symbols. Such a setting nevertheless does not come as a surprise since it emerges naturally already in the context of the Lagrangian EH theory (see previous discussion in Section 2.3 and Section 3.2).

### 4.1. The Proper-Time Parametrization of Space-Time

We start defining the notion of proper time in the context of GR and its physical interpretations. Its geometric definition follows by introducing a mapping between a subset of the real axis I⊆R and the same background spacetime $\left\{Q^{4},\hat{g}\left(r\right)\right\}$ of the form

(81) r=r(s),

being *r* an arbitrary 4-position (“*point*”) of $\left\{Q^{4},\hat{g}\left(r\right)\right\}$ and s∈I⊆R a suitable 4-scalar, to be denote here as “*proper time*”. Therefore, the geometric meaning of *s* depends on its precise prescription. This is obtained by means of two definitions:

(A) *Proper time s*

First, identifying *s* with the arc length $ds^{2}={\hat{g}}_{\mu \nu }dr^{\mu }dr^{\nu }$ measured along an appropriate finite-length geodetic defined with respect to the background space-time $\left\{Q^{4},\hat{g}\left(r\right)\right\}$, that is,

(82) $$C_{\left(r_{o},r_{1}\right)}=\left\{\left.r\right|r=r\left(s^{\prime }\right),r_{o}=r\left(s_{o}\right),r_{1}=r\left(s_{1}\right),s^{\prime }\in \left[s_{o},s_{1}\right],\;r_{o}\in \Sigma_{0}^{3},r_{1}\in \Sigma_{1}^{3}\right\},$$

$\Sigma_{0}^{3}$ and $\Sigma_{1}^{3}$ being two suitable subsets of $Q^{4}$ (see below). Denoting $\frac{dr^{\mu }}{ds}=t^{\mu }\left(r\right)$ the tangent to the curve at *r* and $\nabla_{\mu }$ the covariant derivative evaluated with respect to the background spacetime $\left\{Q^{4},\hat{g}\left(r\right)\right\}$, then by construction along the same curve if follows identically that $\nabla_{\mu }t^{\mu }\left(r\right)=0$.

(B) *Family of geodetics*$\left\{C_{\left(r_{o},r_{1}\right)}\right\}$

The family $\left\{C_{\left(r_{o},r_{1}\right)}\right\}$ is defined in such a way that:

(1) For fixed proper times $s_{o}$ and $s_{1}$ (with $s_{o}<$$s_{1}$), each curve $C_{\left(r_{o},r_{1}\right)}\in \left\{C_{\left(r_{o},r_{1}\right)}\right\}$ has the extrema $r_{o}=r\left(s_{o}\right),r_{1}=r\left(s_{1}\right)$ crossed by the same curve respectively at proper times $s_{o}$ and $s_{1}$. In addition, by assumption: (a) all curves $C_{\left(r_{o},r_{1}\right)}$ belong to the same connected subset $Q_{1}^{4}$ of spacetime $\left\{Q^{4},\hat{g}\left(r\right)\right\}$ which has everywhere the same signature; (b) the lower and upper extrema $r_{o}=r\left(s_{o}\right)$ and $r_{1}=r\left(s_{1}\right)$ belong to two smooth hypersurfaces $\Sigma_{0}^{3}$ (“lower” boundary) and $\Sigma_{1}^{3}$ (“upper” boundary) of the subset of $Q_{1}^{4}$. Hence, $Q_{1}^{4}$ is the subset of $Q^{4}$ having lower and upper boundaries $\Sigma_{0}^{3}$ and $\Sigma_{1}^{3}$ where the extrema of all curves $C_{\left(r_{o},r_{1}\right)}$ lie.

(2) For each point *r* of the said subset of $Q_{1}^{4}$ there is a unique curve $C_{\left(r_{o},r_{1}\right)}\in$$\left\{C_{\left(r_{o},r_{1}\right)}\right\}$ which belongs to it.

(3) Two geodetics of the family never cross each other.

(4) All geodetic curves $C_{\left(r_{o},r_{1}\right)}$ are prescribed in such away to be mono-energetic, that is, so that, in the frame in which $r_{o}=r\left(s_{o}\right)$ the spacetime is locally flat, the corresponding tangent 4-vectors

(83) $$t^{\mu }\left(r_{o}\right)\equiv {\left.\frac{dr^{\mu }}{ds}\right|}_{r_{o}}$$

have all the same zero component of the 4-velocity.

The proper-time parametrization (*s*-parametrization) of spacetime is then realized by parametrizing the background metric tensor $\hat{g}\left(r\right)=\left\{{\hat{g}}_{\mu \nu }\left(r\right)\right\}$ and the canonical tensor fields of the Hamiltonian formulation (see below) $x_{R}=\left\{g_{\mu \nu },\pi^{\mu \nu }\right\}$, and in particular the variational tensor field $g=\left\{g_{\mu \nu }\right\}$, in terms of the proper time *s*. Thus, at the classical level, the *s*-parametrization of $\hat{g}\left(r\right)$ is obtained by means of the representation

(84) $$\hat{g}\left(r\right)\to \hat{g}\left(r\left(s\right)\right)\equiv \left\{{\hat{g}}_{\mu \nu }\left(r\left(s\right)\right)\right\}.$$

Instead, the corresponding parametrization of the variational tensor field $g=\left\{g_{\mu \nu }\right\}$ and its conjugate canonical momentum $\pi =\left\{\pi^{\mu \nu }\right\}$ is obtained letting

(85) g → g(s)≡g(r(s),s),

(86) π → π(s)≡π(r(s),s).

Finally, regarding the physical interpretation, this amounts to assume that the Hamiltonian structure of GR (to be suitably identified) corresponds to disturbances (or signals) of the background metric tensor which propagate in the space-time and occur with finite, that is, subluminal, speed of propagation measured by the proper-time *s*. Such an interpretation will become, nevertheless, obvious in the quantum formulation where such “signals” are interpreted in terms of massive gravitons (see Ref. [29]).

### 4.2. The Manifestly-Covariant Hamiltonian Structure of GR

In this section the manifestly-covariant Hamiltonian structure of GR, that is, associated to EFE and developed in Refs. [8,9,10], is recalled. It realizes in the case of the gravitational field the so-called deDonder-Weyl manifestly-covariant canonical representation for continuum fields [22,23,24,25,26,27,28]. To begin with, we recall some of its peculiar characteristics. The first one is the adoption of independent and symmetric Lagrangian variables $g\equiv \left\{g_{\mu \nu }\right\}\equiv \left\{g_{\nu \mu }\right\}$ associated with the physical properties of the gravitational field. Notice that the tensor *g* is to be distinguished from the background metric tensor $\hat{g}\equiv \left\{{\hat{g}}_{\mu \nu }\right\}$ which determines instead the geometric properties of the space-time and raises/lowers tensor indices [10]. Hereon, for definiteness $\hat{g}$ identifies an in principle arbitrary particular solution of the Einstein field equations, with *g* being an in principle arbitrary and independent real symmetric tensor. As such *g* is assumed to belong by assumption to the functional class ${\left\{g\right\}}_{U}$ defined above (see Equation (36)) and is therefore not required to satisfy orthogonality conditions of the type (5).

Then, according to Ref. [10] the classical Hamiltonian structure of GR is represented by the set $\left\{x_{R},H_{R}\right\}$, formed by the *s*-parametrized canonical state $x_{R}\left(s\right)\equiv \left(g\left(s\right),\pi \left(s\right)\right)$ and a suitable classical Hamiltonian density $H_{R}$. We remark that here the tensor properties of the canonical fields $g\left(s\right)=\left\{g_{\mu \nu }\left(s\right)\right\},\pi \left(s\right)=\left\{\pi^{\mu \nu }\left(s\right)\right\}$ and the Hamiltonian density are prescribed in terms of the background metric tensor $\hat{g}\left(r\left(s\right)\right)$, the first ones being identified with real second order tensor fields and the second one as a real 4-scalar. Then, introducing the Poisson bracket $\left[A,B\right]=\frac{\partial A}{\partial g_{\mu \nu }}\frac{\partial B}{\partial \pi^{\mu \nu }}-\frac{\partial A}{\partial \pi^{\mu \nu }}\frac{\partial B}{\partial g_{\mu \nu }}$, by construction the canonical state $x_{R}\left(s\right)$ fulfills a corresponding set of continuum Hamilton equations

(87) $$\left\{\begin{array}{c} \frac{dg_{\mu \nu }}{ds}=\left[g_{\mu \nu },H_{R}\right]=\frac{\partial H_{R}}{\partial \pi^{\mu \nu }},\\ \frac{d\pi^{\mu \nu }}{ds}=\left[\pi^{\mu \nu },H_{R}\right]=-\frac{\partial H_{R}}{\partial g_{\mu \nu }}, \end{array}\right.$$

which satisfies the initial-value condition

(88) $$x_{R}\left(s_{o}\right)\equiv \left(g_{\mu \nu }\left(s_{o}\right),\pi^{\mu \nu }\left(s_{o}\right)\right),$$

where $g_{\mu \nu }\left(s_{o}\right)$ and $\pi^{\mu \nu }\left(s_{o}\right)$ denote two initial conjugate tensor fields and $s_{o}$ is the initial proper-time. Hereon a solution of the initial value problem (87) and (88) will be identified with the symbol ${\bar{x}}_{R}\left(s\right)$ and referred to as *“extremant” of the Hamiltonian structure*$\left\{x_{R},H_{R}\right\}$. As a consequence, the same equations can be viewed as canonical evolution equations for $x_{R}\left(s\right)$. As such, provided the Hamiltonian density $H_{R}$ is sufficiently regular (see below), the same equations determine uniquely the proper-time evolved canonical state $x_{R}\left(s\right)$ in terms of an (in principle arbitrary) initial condition of the type (88).

Here the notations are standard according to Ref. [30] (see also Refs. [10,29]). Thus, while *s* is—as stated above—the proper-time along an arbitrary field geodetics $C_{\left(r_{o},r_{1}\right)}$ which belongs to r=r(s) at proper-time *s*, the differential operator

(89) $$\frac{d}{ds}={\left.\frac{d}{ds}\right|}_{s}+{\left.\frac{d}{ds}\right|}_{r}$$

identifies the *covariant* *s*-*derivative operator*. In particular, ${\left.\frac{d}{ds}\right|}_{s}\equiv t^{\alpha }{\hat{\nabla }}_{\alpha }$ is the directional covariant derivative, with $t^{\alpha }=\frac{dr^{\alpha }\left(s\right)}{ds}$ being the local tangent to the field geodetics, ${\hat{\nabla }}_{\alpha }$ the covariant derivative defined with respect to the background metric tensor $\hat{g}\left(s\right)$ and ${\left.\frac{d}{ds}\right|}_{r}$ denoting the corresponding covariant *s*-partial derivative prescribed according to the same reference. Furthermore, the Hamiltonian density $H_{R}$ is identified with the function

(90) $$H_{R}\equiv T_{R}\left(\pi ,\hat{g}\right)+V,$$

(91) $$V\left(g,\hat{g},r\left(s\right),s\right)\equiv V_{o}+V_{F},$$

where $T_{R}\left(\pi ,\hat{g}\right)$ and $V(g,\hat{g},r\left(s\right),s)$ denote the effective kinetic and the normalized effective potential densities, with $V_{o}$ and $V_{F}$ being respectively the vacuum and non-vacuum (or external) contributions. Thus, in detail, the first one is by construction a function of the canonical momenta only and takes the form

(92) $$T_{R}\left(\pi ,\hat{g}\right)\equiv \frac{1}{2\alpha L}\pi_{\mu \nu }\pi^{\mu \nu },$$

with α and *L* being suitably-prescribed dimensional constant 4-scalars identified according to the treatment given in Ref. [29]. The prescription of the vacuum potential $V_{o}$ follows from analogous one obtained in the case of the asynchronous unconstrained Lagrangian variational principle (44). This implies for the Ricci tensor to be considered extremal, that is, of the form (39) and (40) respectively for its covariant and counter-variant components. The corresponding representation for $V_{o}$ is therefore given by

(93) $$V_{o}\left(g,\hat{g},s\right)\equiv h\alpha L\left(g^{\mu \nu }{\hat{R}}_{\mu \nu }-2\Lambda \right).$$

Here, Λ identifies the cosmological constant and $h(g,\hat{g})$ is a suitable 4-scalar variational weight-factor defined as

$$h=2-\frac{1}{4}g^{\alpha \beta }g^{\mu \nu }{\hat{g}}_{\alpha \mu }{\hat{g}}_{\beta \nu }$$

and finally all hatted quantities, and in particular the Ricci tensor ${\hat{R}}_{\mu \nu }$, are intended as evaluated in terms of the background field tensor $\hat{g}$. Thus, explicit evaluation on the rhs of Equation (87) yields

(94) $$\left\{\begin{array}{c} \frac{dg_{\mu \nu }}{ds}=\frac{\pi_{\mu \nu }}{\alpha L},\\ \frac{d\pi^{\mu \nu }}{ds}=-\frac{\partial V}{\partial g_{\mu \nu }}, \end{array}\right.$$

where the first equation prescribes the canonical momentum $\pi_{\mu \nu }$ in terms of the “generalized velocity” $\frac{dg_{\mu \nu }}{ds}$, while the second one provides a dynamical equation for the canonical momentum. The connection with EFE follows in straightforward way by setting the initial condition

(95) $$x_{R}\left(s_{o}\right)=\left(g_{\mu \nu }\left(s_{o}\right)\equiv {\hat{g}}_{\mu \nu }\left(s_{o}\right),\pi^{\mu \nu }\left(s_{o}\right)\equiv {\hat{\pi }}^{\mu \nu }\left(s_{o}\right)=0\right),$$

with ${\hat{g}}_{\mu \nu }\left(s_{o}\right)$ denoting the background metric tensor evaluated at $s_{o}$. In fact, the initial condition ${\bar{x}}_{R}\left(s_{o}\right)\equiv x_{R}\left(s_{o}\right)$ must necessarily be such that

(96) $${\left.\frac{\partial V(g,r(s),s)}{\partial g_{\mu \nu }}\right|}_{g=\hat{g},s=s_{o}}=0,$$

with ${\hat{\pi }}^{\mu \nu }\left(s_{o}\right)=0$ being the corresponding initial null canonical momentum. Then, for all $s>s_{o}$, $x_{R}\left(s\right)=\left(g_{\mu \nu }\left(s\right)\equiv {\hat{g}}_{\mu \nu }\left(s\right),\pi^{\mu \nu }\left(s\right)\equiv {\hat{\pi }}^{\mu \nu }\left(s\right)\equiv 0\right)$, with ${\hat{g}}_{\mu \nu }\left(s\right)$ being for all (r(s),s) solution of the stationary equation:

(97) $${\left.\frac{\partial V(g,r(s),s)}{\partial g_{\mu \nu }}\right|}_{g=\hat{g}}=0.$$

Indeed, the initial conditions (95) together with Equation (96) imply that ${\left.\frac{d\pi^{\mu \nu }}{ds}\right|}_{s=s_{o}}=0$.

The proof is straightforward. First, we notice that Equation (96) necessarily follows by setting the initial condition (95). In fact, the same initial condition $g\left(s_{o}\right)\equiv \hat{g}\left(s_{o}\right)$ requires that $g(s_{o})$ must be a solution of EFE (see, e.g., Equation (5)), while on the other hand elementary algebra shows that Equation (96) necessarily coincides with

(98) $${\hat{R}}_{\mu \nu }\left(s_{o}\right)-\frac{1}{2}\hat{R}\left(s_{o}\right){\hat{g}}_{\mu \nu }\left(s_{o}\right)+\Lambda {\hat{g}}_{\mu \nu }\left(s_{o}\right)=\frac{8\pi G}{c^{4}}{\hat{T}}_{\mu \nu }\left(s_{o}\right),$$

namely EFE evaluated at $r\left(s_{o}\right)=r_{o}$ along the geodesic trajectory $C_{\left(r_{o},r_{1}\right)}$. As a consequence, it follows by construction also that ${\left.\frac{d\pi^{\mu \nu }}{ds}\right|}_{s_{o}}=0$. Hence, this means that $\pi^{\mu \nu }\left(s\right)$ is stationary and null also for $s>s_{o}$, while $g_{\mu \nu }\left(s\right)$ remains stationary too, being therefore determined by Equation (97) which manifestly coincides with

(99) $${\hat{R}}_{\mu \nu }\left(s\right)-\frac{1}{2}\hat{R}\left(s\right){\hat{g}}_{\mu \nu }\left(s\right)+\Lambda {\hat{g}}_{\mu \nu }\left(s\right)=\frac{8\pi G}{c^{4}}{\hat{T}}_{\mu \nu }\left(s\right),$$

that is, nothing else than EFE evaluated at the proper time *s*, and again along the same geodesic trajectory $C_{\left(r_{o},r_{1}\right)}$.

As a consequence, it is concluded that the canonical Equation (87) indeed recover EFE upon setting the appropriate initial conditions (95). More precisely, comparison with the ADM approach to the Hamiltonian theory of GR permits to establish the following features:

- The evolution Equations (87) and (88) are uniquely associated to EFE. In fact, the same equations reduce to EFE by prescribing appropriate initial conditions in which the initial canonical momentum vanishes. For the initial condition (95) such a solution is unique. In addition the same equations are truly Hamiltonian in character. In fact the evolution Equations (87) and (88) define a true Hamiltonian system because, unlike the ADM approach, no unwanted hidden non-local dependences (which actually would destroy the same Hamiltonian structure) appear in the Hamiltonian density. Basic consequence is therefore that the set $\left\{x_{R},H_{R}\right\}$ can be identified as a possible candidate for the Hamiltonian structure of GR.
- The Hamiltonian system $\left\{x_{R},H_{R}\right\}$ is constraint-free because, in contrast to the ADM approach, the tensor components of the Hamiltonian state $x_{R}\left(s\right)\equiv \left(g\left(s\right),\pi \left(s\right)\right)$ are independent. In fact, the requirement which permits to recover EFE is merely the natural dynamical constraint which arises due to the initial conditions. In addition, no boundary constraint conditions are set in such a framework. The same Hamilton structure, unlike in the ADM approach, is preserved also in validity of the initial condition (95) which actually permits to recover EFE.
- Finally, the most important feature which emerges in a perspicuous way by inspection of Equations (87) and (88) is that the Hamiltonian system $\left\{x_{R},H_{R}\right\}$ is manifestly covariant, that is, it is set in 4-tensor form, since both $x_{R}$ and $H_{R}$ have a 4-tensor and therefore frame-independent character with respect to the background spacetime $\left\{Q^{4},\hat{g}\left(r\right)\right\}$.

### 4.3. Synchronous Path-Integral Hamiltonian Variational Principle

Let us now address the issue of casting the continuous Hamilton Equation (94) in variational form, that is, in terms of a suitable realization of the corresponding variational principle. For its close similarity with the standard theory of Hamiltonian dynamical systems, an obvious answer to this question is provided by what can be referred to as a *Hamiltonian variational principle*. Let us introduce for this purpose the path-integral Hamiltonian functional (see also Ref. [31])

(100) $$J\left(x_{R}\left(s\right)\right)=\int_{s_{o}}^{s_{1}}dsL_{R}\left(g\left(s\right),\frac{dg\left(s\right)}{ds},s\right),$$

where

(101) $$L_{R}\left(g\left(s\right),\frac{dg\left(s\right)}{ds},s\right)=\pi^{\mu \nu }\left(s\right)\frac{dg_{\mu \nu }\left(s\right)}{ds}-H_{R}\left(x_{R}\left(s\right),s\right),$$

and $\frac{d}{ds}$ is the covariant derivative (89) acting on a tensor function of the form $f_{\mu \nu }\left(r\left(s\right),s\right)$, which is parametrized as $\frac{d}{ds}=$$t^{\alpha }\left(s\right){\hat{\nabla }}_{\alpha }$$+\frac{\partial }{\partial s}$. Furthermore, here $L_{R}\left(g\left(s\right),\frac{dg\left(s\right)}{ds},s\right)$ and $H_{R}\left(x_{R}\left(s\right),s\right)$ are respectively the Lagrangian and the Legendre-conjugate Hamiltonian densities, whereas the canonical state $x_{R}$, represented in terms of its *s*-parametrization $x_{R}\left(s\right)=\left(g_{\mu \nu }\left(s\right),\pi^{\mu \nu }\left(s\right)\right)$, belongs by assumption to the synchronous functional class

(102) $${\left\{x_{R}\left(s\right)\right\}}_{S}=\left\{\left.x_{R}\right|x_{R}\left(s\right)={\bar{x}}_{R}\left(s\right)+\delta x_{R}\left(s\right),x_{R}\left(s_{i}\right)=x_{Ri},x_{Ri}\in \Sigma_{i}^{3},i=0,1\right\}.$$

Here ${\bar{x}}_{R}\left(s\right)$ denotes the general solution of the initial value problem (87) and (88), that is, a so-called *extremant of the Hamiltonian structure*$\left\{x_{R},H_{R}\right\}$, while the boundary conditions $x_{R}\left(s_{1}\right)$ is not considered independent of $x_{R}\left(s_{o}\right)$. Finally, here $\delta x_{R}\left(s\right)$ denotes the synchronous variation, that is, an arbitrary real tensor function $\delta x_{R}\left(s\right)\equiv \left(\delta g_{\mu \nu }\left(s\right),\delta \pi^{\mu \nu }\left(s\right)\right)$ such that

(103) $$\delta x_{R}\left(s_{i}\right)=0$$

for $s=s_{o}$ and $s_{1}$, while

(104) δs=0

for all $s\in \left[s_{o},s_{1}\right]$. The connection with the canonical Equation (87) can then be established at once, as it follows from the Hamiltonian variational principle

(105) $$\delta J(x_{R}\left(s\right))=0,$$

where the variation operator δ denotes again the Frechet derivative and the variations $\delta x_{R}\left(s\right)$ are considered arbitrary and independent, namely

(106) $$\delta J\left(x_{R}\right)\equiv {\left.\frac{d}{d\alpha }J\left({\bar{x}}_{R}\left(s\right)+\alpha \delta x_{R}\left(s\right)\right)\right|}_{\alpha =0}.$$

For the precise evaluation of (105) one notices that by construction the general solution ${\bar{x}}_{R}\left(s\right)$ is independent of the parameter α. Hence, its synchronous variation necessarily vanishes identically, so that $\delta {\bar{x}}_{R}\left(s\right)\equiv {\left.\frac{d}{d\alpha }{\bar{x}}_{R}\left(s\right)\right|}_{\alpha =0}\equiv 0$. As a consequence, also for the particular solution represented by the background metric tensor $\hat{g}\left(s\right)$ the corresponding synchronous variation is vanishing identically, that is, $\delta \hat{g}\left(s\right)\equiv 0$. The establishment of the corresponding Euler-Lagrange equations follows in terms of the functional derivatives of $J(x_{R}\left(s\right))$:

(107) $$\left\{\begin{array}{c} \frac{\delta J(x_{R}\left(s\right))}{\delta \pi^{\mu \nu }\left(s\right)}=0,\\ \frac{\delta J(x_{R}\left(s\right))}{\delta g_{\mu \nu }\left(s\right)}=0. \end{array}\right.$$

For this purpose one notices that the following differential identity applies

(108) $$\pi^{\mu \nu }\left(s\right)\frac{dg_{\mu \nu }\left(s\right)}{ds}=\frac{d}{ds}\left[\pi^{\mu \nu }\left(s\right)g_{\mu \nu }\left(s\right)\right]-g_{\mu \nu }\left(s\right)\frac{d\pi^{\mu \nu }\left(s\right)}{ds},$$

which implies that

(109) $$\int_{s_{o}}^{s_{1}}ds\frac{d}{ds}\left[\pi^{\mu \nu }\left(s\right)g_{\mu \nu }\left(s\right)\right]={\left.\pi^{\mu \nu }\left(s\right)g_{\mu \nu }\left(s\right)\right|}_{s_{1}}^{s_{1}}$$

is a gauge contribution (to the variational functional $J(x_{R})$) which, by construction (i.e., thanks to the prescription of the synchronous functional class ${\left\{x_{R}\left(s\right)\right\}}_{S}$), does not contribute. As a result, the corresponding Euler-Lagrange equations follow at once, being identical to the Hamilton equations indicated above (see Equation (87)) and with solutions subject to the boundary conditions $x_{R}\left(s_{i}\right)=x_{Ri}$ (for i=0,1). The latter ones can then be shown to be necessarily equivalent to (88). However, from direct inspection of the path-integral Hamiltonian variational principle (105), a number of consequences follow which are unprecedented, especially in connection with the ADM theory. More precisely these are represented by the following additional notable features:

- The path-integral Hamiltonian variational principle is set in a manifestly-covariant form.
- The path integral in the functional $J(x_{R})$ is performed along a generic finite-length field geodetics $C_{\left(r_{o},r_{1}\right)}$ so that $x_{R}\left(s\right)$ is parametrized in terms of it, namely letting $x_{R}\left(s\right)\equiv x_{R}\left(r(s),s\right)$ while the Hamiltonian density is of the general form $H_{R}\equiv H_{R}\left(x_{R}\left(s\right),r\left(s\right),s\right)$.
- The tensor components of the variation $\delta x_{R}$ are considered independent. For this reason the variational principle (105) can be referred to as constraint-free.
- The variation operator δ in the path-integral variational principle defined above (106) is *synchronous*, that is, it is such that it leaves invariant both the proper time *s* and the field geodetics $C_{\left(r_{o},r_{1}\right)}$ so that identically
  (110) $$\left\{\begin{array}{c} \delta (ds)\equiv 0\\ \delta r\left(s\right)=0 \end{array}\right.,$$
  and furthermore, by construction, it leaves invariant also the corresponding boundary conditions, so that
  (111) $$\delta x_{R}\left(s_{o}\right)=\delta x_{R}\left(s_{1}\right)=0.$$
- Both the Hamiltonian and Lagrangian densities $H_{R}\left(x_{R}\left(s\right),s\right)$ and $L_{R}\left(g\left(s\right),\frac{dg\left(s\right)}{ds},s\right)$ are intrinsically non-unique, being necessarily determined up to an additive gauge function of the form
  (112) $$\frac{d}{ds}F\left(g\left(s\right),r\left(s\right),s\right),$$
  with $F(g\left(s\right),s)$ denoting a real arbitrary 4-scalar field of class $C^{\left(2\right)}$. In fact, the term
  (113) $$\Delta J\left(x_{R}\left(s\right)\right)=\int_{s_{o}}^{s_{1}}ds\frac{d}{ds}F\left(g\left(s\right),r\left(s\right),s\right)\equiv {\left.F(g\left(s\right),r\left(s\right),s)\right|}_{s_{o}}^{s_{1}}$$
  has by construction null variation since
  (114) $$\delta \left\{{\left.F(g\left(s\right),r\left(s\right),s)\right|}_{s_{o}}^{s_{1}}\right\}\equiv 0.$$
  As a consequence, the path-integral Hamiltonian variational principle (105) is gauge invariant.

### 4.4. A Hypersurface-Integral Hamiltonian Variational Principle

The variational principle reported above, that is, Equation (105), applies to a single (albeit arbitrary) geodesic trajectory $C_{\left(r_{o},r_{1}\right)}$. However, the extension to include the whole set of trajectories $\left\{C_{\left(r_{o},r_{1}\right)}\right\}$ is actually obvious. In fact, the family of geodesic curves $\left\{C_{\left(r_{o},r_{1}\right)}\right\}$ of $\left\{Q^{4},\hat{g}\left(r\right)\right\}$ generates by assumption for all $s\in \left[s_{o},s_{1}\right]$ and all $r_{o}\in \Sigma_{0}^{3}\subset Q_{1}^{4}\subseteq Q^{4}$ a flow, that is, a map

(115) $$r_{o}=r\left(s_{o}\right)\to r=r\left(s\right)\equiv \chi \left(r_{o},s_{o},s\right),$$

which is in turn generated by a classical dynamical system (i.e., a bijection). Thus, the path integral functional (63) can be integrated on the set $\Sigma_{0}^{3}$ (a 3D subset of $Q_{1}^{4}\subseteq Q^{4}$, that is, the hypersurface on which $s=s_{o}$) yielding

(116) $$S\left(x_{R}\right)=\int_{s_{o}}^{s_{1}}ds\int_{Q^{4}}d\Omega_{o}\delta_{\Sigma^{3}}\left(r_{o}\right)\left\{\pi^{\mu \nu }\left(s\right)\frac{dg_{\mu \nu }\left(s\right)}{ds}-H_{R}\left(x_{R}\left(s\right),s\right)\right\},$$

which is now defined with respect to the functional class

(117) $${\left\{x_{R}\left(s\right)\right\}}_{\Sigma^{3}}=\left\{\left.x_{R}\right|x_{R}\left(s\right)={\bar{x}}_{R}\left(s\right)+\delta x_{R}\left(s\right),x_{R}\left(s_{i}\right)=x_{Ri},x_{Ri}\in \Sigma_{i}^{3},i=0,1\right\},$$

where ${\bar{x}}_{R}\left(s\right)\equiv \left\{{\bar{g}}_{\mu \nu }\left(s\right),{\bar{\pi }}^{\mu \nu }\left(s\right)\right\}$ denotes the general solution of the Hamiltonian Equation (107) (see equivalently Equation (120) below), which includes as a particular solution also $\hat{g}\left(s\right)\equiv \left\{{\hat{g}}_{\mu \nu }\left(s\right)\right\}$. Furthermore, the tensor variation $\delta x_{R}\left(s\right)$ is assumed once again smooth and everywhere bounded for all $s\in \left[s_{o},s_{1}\right]$ so that again Equations (110) and (111) hold. Here furthermore

(118) $$d\Omega_{o}=d^{4}r_{o}\sqrt{-\left|\hat{g}\left(r_{o}\right)\right|}$$

denotes the invariant volume element of the spacetime $\left\{Q^{4},\hat{g}\left(r\right)\right\}$, and therefore evaluated with respect to the background metric tensor at the position $r_{o}$. As a consequence, by construction manifestly $\delta d\Omega_{o}=0$. Moreover, $\delta_{\Sigma^{3}}\left(r_{o}\right)$ is the characteristic function on the ensemble $\Sigma^{3}$, that is, defined so that $\delta_{\Sigma_{0}^{3}}\left(r_{o}\right)=1$ if $r_{o}\in \Sigma_{0}^{3}$ and 0 otherwise. We stress that in the integrand the canonical state $x_{R}\left(s\right)\equiv \left\{g_{\mu \nu }\left(s\right),\pi^{\mu \nu }\left(s\right)\right\}$ is considered a composite function of $r_{o}$ through $r\left(s\right)=\chi \left(r_{o},s_{o},s\right)$. Thus, ${\bar{x}}_{R}\left(s\right)$, $\hat{g}\left(s\right)$, $g_{\mu \nu }\left(s\right)$ (and similarly $\pi^{\mu \nu }\left(s\right)$) are treated as functions of $r_{o}$ since ${\hat{g}}_{\mu \nu }\left(s\right)\equiv {\hat{g}}_{\mu \nu }\left(r\left(s\right)=\chi \left(r_{o},s_{o},s\right)\right)$ and respectively $g_{\mu \nu }\left(s\right)\equiv g_{\mu \nu }\left(r\left(s\right)=\chi \left(r_{o},s_{o},s\right),s\right)$.

Once again the connection with the canonical Equation (87) can be formally established just as in the path-integral approach. This follows of course from the (volume-integral) synchronous Hamiltonian variational principle

(119) $$\delta S\left(x_{R}\right)\equiv {\left.\frac{d}{d\alpha }S\left({\bar{x}}_{R}\left(s\right)+\alpha \delta x_{R}\left(s\right)\right)\right|}_{\alpha =0}=0,$$

the components of the tensor variations $\delta x_{R}\left(s\right)$ being arbitrary and independent also in this case. Furthermore, again the same considerations indicated above (see Section 4.3) apply here regarding the synchronous variations of ${\bar{x}}_{R}\left(s\right)$ and $\hat{g}\left(s\right)$. The corresponding symbolic Euler-Lagrange equations given by the variational derivatives then coincide with the canonical Equation (87)

(120) $$\left\{\begin{array}{c} \frac{\delta S(x_{R}\left(s\right))}{\delta \pi^{\mu \nu }\left(s\right)}=\frac{dg_{\mu \nu }}{ds}-\frac{\partial T_{R}}{\partial \pi^{\mu \nu }}=0,\\ \frac{\delta S(x_{R}\left(s\right))}{\delta g_{\mu \nu }\left(s\right)}=-\frac{d\pi^{\mu \nu }}{ds}-\frac{\partial V}{\partial g_{\mu \nu }}=0, \end{array}\right.$$

which are subject (again) only to the initial conditions (88).

The variational principle (119) is obviously not independent from the one given above in term of the path-integral (100). So, the same conclusions discussed above actually apply. Nevertheless, additional peculiar aspects can be inferred. The first one concerns the synchronous character of the volume-integral variational principle. We stress in fact that the variation operator δ which appears in the variational principle (119) is again of the synchronous type. In fact, besides leaving invariant the proper time element ds and the proper time *s*, it leaves invariant by construction also the volume element $d\Omega_{o}$. As a further remark, however, we also notice that the same operator δ leaves invariant by construction also the generic volume element centered at r=r(s), namely $d\Omega \left(s\right)=d^{4}r\sqrt{-\left|\hat{g}\left(r\left(s\right)\right)\right|}$, because $\delta \hat{g}\left(r\right)=\delta r^{\mu }\nabla_{\mu }$$\hat{g}\left(r\right)$ and, again by construction, $\nabla_{\mu }$$\hat{g}\left(r\right)\equiv 0$ since the Christoffel symbols in the covariant derivative are by assumption defined with respect to the same $\hat{g}\left(r\right)$. Finally, in the functional (116) the integration is actually carried out on a 3-dimensional subset of spacetime, that is, a hypersurface $\Sigma_{0}^{3}$. This feature seems interesting because it may be suggestive of its possible physical interpretation as a “source” of the geodetics.

### 4.5. A Volume-Integral Hamiltonian Variational Principle

An equivalent realization of the variational principle, alternative to those reported above, that is, (105) and (119), can be achieved upon parametrizing the canonical state as $x_{R}\left(r,s\right)=\left\{g_{\mu \nu }\left(r,s\right),\pi^{\mu \nu }\left(r,s\right)\right\}$. As a result, the Lagrangian coordinates and canonical momentum now become of the form

(121) $$\left\{\begin{array}{c} g_{\mu \nu }\left(r,s\right)={\bar{g}}_{\mu \nu }\left(r,s\right)+\delta g_{\mu \nu }\left(r,s\right),\\ \pi^{\mu \nu }\left(r,s\right)={\bar{\pi }}^{\mu \nu }\left(r,s\right)+\delta \pi^{\mu \nu }\left(r,s\right). \end{array}\right.$$

Thus, denoting by $\partial Q_{1}^{4}$ the boundary of $Q_{1}^{4}$, the following functional setting is adopted

(122) $$\begin{array}{ccc} & & \left|{\left\{x_{R}\left(r,s\right)\right\}}_{Q_{1}^{4}}=\right|\\ & & \left\{\left.x_{R}\right|x_{R}\left(r,s\right)={\bar{x}}_{R}\left(r\right)+\delta x_{R}\left(r,s\right),{\left.\delta x_{R}\left(r,s\right)\right|}_{r\in \partial Q_{1}^{4}}=0,\delta x_{R}\left(r,s_{i}\right)=0,i=0,1\right\}, \end{array}$$

with $\delta x_{R}\left(r,s\right)$ being assumed again smooth and everywhere bounded on $Q_{1}^{4}$ and the boundary conditions on $\partial Q_{1}^{4}$ being set so that

(123) $$\left\{\begin{array}{c} {\left.\delta g_{\mu \nu }\left(r,s\right)\right|}_{r\in \partial Q_{1}^{4}}=0,\\ {\left.\delta \pi^{\mu \nu }\left(r,s\right)\right|}_{r\in \partial Q_{1}^{4}}=0. \end{array}\right.$$

In this case the relevant variational functional can be identified with the action functional

(124) $$Q\left(x_{R}\left(r,s\right)\right)=\frac{1}{\int_{s_{o}}^{s_{1}}ds}\int_{s_{o}}^{s_{1}}ds\int_{Q_{1}^{4}}d\Omega \left\{\pi^{\mu \nu }\left(r,s\right)\frac{dg_{\mu \nu }\left(r,s\right)}{ds}-H_{R}\left(x_{R}\left(r,s\right),s\right)\right\},$$

with dΩ being the volume element evaluated with respect to the background spacetime, that is, $d\Omega =d^{4}r\sqrt{-\left|\hat{g}\left(r\right)\right|}$, and the covariant derivative being prescribed according to Equation (89). Notice that in the functional setting (122) the tensor variation $\delta x_{R}\left(r,s\right)$ are required to be continuous so that in accordance with the boundary conditions (123), $\delta x_{R}\left(r,s\right)$ must tend smoothly to zero on the boundary $\partial Q_{1}^{4}$. This implies therefore by construction that $\delta \int_{Q_{1}^{4}}d\Omega t^{\alpha }\left(r\right)\nabla_{\alpha }\left[g_{\mu \nu }\left(r,s\right)\pi^{\mu \nu }\left(r,s\right)\right]=0$ so that the following identity

(125) $$\begin{array}{ccc} & & \delta \int_{s_{o}}^{s_{1}}ds\int_{Q_{1}^{4}}d\Omega \left[\pi^{\mu \nu }\left(r,s\right)\frac{dg_{\mu \nu }\left(r,s\right)}{ds}+g_{\mu \nu }\left(r,s\right)\frac{d\pi^{\mu \nu }\left(r,s\right)}{ds}\right]\\ & & \left.=\delta \int_{Q_{1}^{4}}d\Omega {\left[{\bar{\pi }}^{\mu \nu }\left(r,s\right){\bar{g}}_{\mu \nu }\left(r,s\right)\right]}_{s_{o}}^{s_{1}}=0\right. \end{array}$$

necessarily holds. Then, straightforward algebra displays the connection with the canonical Equation (87). This follows upon invoking that the synchronous variational principle

(126) $$\delta Q\left(x_{R}\left(r,s\right)\right)\equiv {\left.\frac{d}{d\alpha }Q\left({\bar{x}}_{R}\left(r,s\right)+\alpha \delta x_{R}\left(r,s\right)\right)\right|}_{\alpha =0}=0$$

holds for arbitrary independent variations $\delta x_{R}\left(r,s\right)=\left\{\delta g_{\mu \nu }\left(r,s\right),\delta \pi^{\mu \nu }\left(r,s\right)\right\}$ and the same considerations indicated above (see Section 4.3) apply to the synchronous variations of the general solution ${\bar{x}}_{R}\left(s\right)$ and to the particular solution realized by $\hat{g}\left(s\right)$. Here we notice again that the variation operator is synchronous in the sense that, besides Equations (110) and (111), also the requirement δdΩ=0 holds. Then, the corresponding Euler-Lagrange equations, namely

(127) $$\left\{\begin{array}{c} \frac{\delta JQ(x_{R}\left(r,s\right))}{\delta \pi^{\mu \nu }\left(r,s\right)}=0,\\ \frac{\delta Q(x_{R}\left(r,s\right))}{\delta g_{\mu \nu }\left(r,s\right)}=0, \end{array}\right.$$

can be shown to coincide with Equation (87). We remark, finally, that in the case the canonical state is assumed not to depend explicitly on *s*, then the action functional (124) simply reduces to

(128) $$Q\left(x_{R}\left(r\right)\right)=\int_{Q_{1}^{4}}d\Omega \left\{\pi^{\mu \nu }\left(r\right)\frac{dg_{\mu \nu }\left(r,s\right)}{ds}-H_{R}\left(x_{R}\left(r\right)\right)\right\},$$

where the Lagrangian derivative is now identified with the directional covariant derivative $\frac{d}{ds}=t^{\alpha }\left(r\right)\nabla_{\alpha }$, that is, the covariant derivative projected along the tangent to the local geodesic curve belonging to the family $\left\{C_{r_{o}},r_{1}\right\}$.

## 5. Physical Interpretation

The results presented above permit to clarify the conditions of validity and limits of previous approaches to the Lagrangian EH and Hamiltonian ADM variational principles. The main conclusions are that, with the possible exception of the Palatini approach, some of the previous approaches to the Lagrangian EH theory appear questionable. As far as Hamiltonian theory is concerned, we have shown that the attempt of identifying the ADM variables in terms of true canonical variables fails because of the intrinsic non-Hamiltonian character of the corresponding non-manifestly covariant theory. The reason of the failure is that the Ricci 4-scalar unavoidably carries second-order partial derivative of the same variables, a feature which destroys their Hamiltonian character.

The way-out solution for the Lagrangian approaches to GR is represented respectively by the unconstrained Lagrangian variational principle (44) and the unconstrained Lagrangian variational formulation for the ADM representation (74). Instead, the way-out solution for the Hamiltonian approach is given respectively by the three synchronous Hamiltonian variational principles represented by the path-integral Hamiltonian variational principle (105), the hypersurface-integral Hamiltonian variational principle (119) and the volume-integral Hamiltonian variational principle (126).

We stress that these variational formulations are based on the adoption of constraint-free variational principles together with a suitable prescription of the functional setting. For this purpose the Ricci tensor is considered prescribed in terms of a suitable background metric tensor $\hat{g}\left(r\right)$, to be identified with a particular solution of EFE, which raises and lowers all tensor indices and determines the geometry of spacetime. This means, more precisely, that in all variational principles referred to above, the contribution $g_{\mu \nu }{\hat{R}}^{\mu \nu }$ enters the Lagrangian density. The same term, in fact, gives rise to a well-defined contribution to the effective potential energy (see Equation (91) above) which occurs both in the Lagrangian and Hamiltonian densities.

Let us now show how this contribution can be given a well-defined physical interpretations. We first notice that, according to Equations (39) and (40), in the same variational principles ${\hat{R}}^{\mu \nu }$ is considered prescribed and independent of the variational field g(r), while $g_{\mu \nu }$ is variational. As a consequence, ${\hat{R}}^{\mu \nu }$ enters as a linear contribution to the Lagrangian and Hamiltonian densities defined above. Furthermore, in $g_{\mu \nu }{\hat{R}}^{\mu \nu }$ both terms $g_{\mu \nu }$ and ${\hat{R}}^{\mu \nu }$ have a tensor character so that the same can be equivalently represented as

(129) $$g_{\mu \nu }{\hat{R}}^{\mu \nu }\equiv g^{\mu \nu }{\hat{R}}_{\mu \nu }.$$

This means that $g_{\mu \nu }{\hat{R}}^{\mu \nu }$ retains a tensorial character since it behaves as a 4-scalar, a feature that confirms its physically objective character. As a consequence its possible physical interpretation follows as a *gravitational coupling term.*

That such an interpretation is appropriate is also suggested by analogy with the variational principle of Maxwell equations where the so-called *EM coupling term* $A_{\mu }J^{\mu }$ occurs. Its properties are similar. In fact, in the case of the EM variational principle $J^{\mu }$ and $A_{\mu }$ are considered respectively prescribed and variational tensor fields, thus warranting the 4-scalar character of the EM coupling term. Moreover, the fact that $J^{\mu }$ is considered constrained means that it is necessarily independent of $A_{\mu }$, so that the coupling term provides a linear relationship in which only the linear term $A_{\mu }$ is involved in the variations of the field, while $J^{\mu }$ enters linearly the extremal Maxwell equations.

Finally, the fact that the Ricci scalar enters the Lagrangian density function as a coupling term has another interesting physical implication. Since it carries only a linear contribution of the metric tensor, namely the field coordinate, and not of the corresponding generalized velocities $\frac{dg_{\mu \nu }}{ds}$, it follows that it can be interpreted as a potential term in the Lagrangian (and Hamiltonian) densities. In other words, it contributes only to the effective potential $V(g,\hat{g},r\left(s\right),s)$ (see Equation (91) and not to the corresponding effective kinetic energy $T_{R}\left(\pi ,\hat{g}\right)$ (90).

The conclusion which one infers is therefore the following one: it appears conceptually incorrect to break the manifest covariance character of the Ricci scalar and the Ricci tensor and proceed—as done in ADM theory—by attempting to construct a Hamiltonian theory of GR in terms of non-tensorial terms which appear in the definition of the Ricci tensor. The same non-tensorial terms, besides breaking the manifest-covariance property, retain in fact quadratic partial-derivative terms and second-order derivatives which give rise to the intrinsically non-Hamiltonian character of ADM theory.

Instead, one envisages a manifestly covariant Hamiltonian theory of GR in which the Ricci tensor is set in its extremal form, acting effectively as a classical “potential” via a gravitational coupling term for an extended Lagrangian function. It is therefore not surprising that the abstract Hamiltonian structure of GR pointed out here, and represented by the set $\left\{x_{R},H_{R}\right\}$, exhibits the standard canonical structure of classical mechanics and classical field theory.

## 6. Conclusions

In this paper, aspects of the variational theory of GR have been investigated. As far as the Lagrangian theory of GR is concerned, based on the Einstein-Hilbert action functional (as discussed in Section 2), the existence of a novel unconstrained Lagrangian variational principle has been pointed out (see Section 2.3 and Section 2.4). Such a variational principle represented in ADM variables (see Section 3.2) has also implications for the physical interpretation and understanding of ADM theory.

The main emphasis has been devoted to the two alternative Hamiltonian representations of GR, here analyzed and compared in detail: namely the ADM and the manifestly-covariant Hamiltonian approaches respectively. The outcome in our view is of potential value for unveiling and weighting the possible physical relevance of the two Hamiltonian representations, also in view of the corresponding quantum formulations. For this purpose the main features, analogies and differences arising between them have been pointed out.

Thus, the ADM theory has been shown to exhibit a number of major deficiencies in particular:

- The structure of the ADM dynamical equations is not truly Hamiltonian, because the same equations still depend on first and second-order spatial partial derivatives of the relevant non-tensor variational fields. The same dynamical equations may not generally satisfy the variational functional characteristic constraint condition of EH theory pointed out here. We refer in particular to the requirement that the Ricci tensor components are functions of the background metric tensor $\hat{g}\left(r\right)$. We have shown in fact that the same Hamilton-like structure is not preserved when such a constraint condition is taken into account.
- ADM theory is not gauge-invariant, a property which is closely related to the nature of the asynchronous variational principle required for its construction.
- Despite being covariant with respect to an arbitrary local point transformation, ADM theory is not manifestly-covariant.

On the contrary, in radical departure from such a complex and even possibly fundamentally incorrect picture, the manifestly-covariant Hamiltonian theory pointed out here presents itself as a straightforward alternative. With a number of notable features, in particular:

- The relevant dynamical equations for the tensor fields are realized by means of evolution-type ODEs which identify a Hamiltonian system in a proper sense. Such a feature is warranted by the fulfillment of the constraint condition characteristic of the Einstein field equations, namely the requirement that the components of the Ricci tensor depend on the background metric tensor $\hat{g}\left(r\right)$ only.
- The corresponding Hamiltonian system $\left\{x_{R},H_{R}\right\}$ is constraint-free because, in contrast to the ADM approach, the tensor components of the Hamiltonian state $x_{R}\left(s\right)\equiv \left(g\left(s\right),\pi \left(s\right)\right)$ are independent.
- The Einstein field equations are determined in terms of a particular solution of the initial-value problem associated with suitable Hamilton equations for the relevant 4-tensor fields. The solution corresponding to such an initial condition, requiring the initial canonical momentum to vanish, is unique. The same Hamiltonian structure, unlike in the ADM approach, is preserved also in validity of the initial condition which actually permits to recover the Einstein field equations.
- The Hamilton equations are determined via a synchronous variational principle which leaves invariant both the proper-time which parametrizes the same equations and the 4-scalar volume element.
- The theory is gauge invariant. As a consequence both the Hamiltonian and Lagrangian densities are intrinsically non-unique, being determined up to an additive gauge function.
- The Hamiltonian system $\left\{x_{R},H_{R}\right\}$ is manifestly covariant, that is, it is set in 4-tensor form, since both $x_{R}$ and $H_{R}$ have a 4-tensor and the frame-independent character with respect to the background spacetime $\left\{Q^{4},\hat{g}\left(r\right)\right\}$.

To conclude, let us summarize the physical interpretation of the manifestly-covariant Hamiltonian structure of GR determined above and how it compares to the ADM theory. The first striking difference is that the manifestly-covariant approach truly realizes a Hamiltonian structure. In fact in such a context the non-local dependencies characteristic of ADM theory, which make it intrinsically non-Hamiltonian, are not extant any more. The ADM dynamical structure appears as a property of the representation of the Ricci tensor, where ${\overset{\cdot }{h}}_{ab}$ is identified with the generalized velocity, but this does not define a true symplectic structure of GR. Curiously, the claimed ADM Hamiltonian structure is generated by the Ricci tensor $R^{\mu \nu }$ that appears coupled to the variational tensor field $g_{\mu \nu }$ in the EH Lagrangian, even though it is proved that the variations of $R^{\mu \nu }$ should not actually contribute to EFE. The reason why the ADM velocity ${\overset{\cdot }{h}}_{ab}$ appears is because $R^{\mu \nu }$ is coupled to $g_{\mu \nu }$ in the Lagrangian. Therefore, the ADM dynamical theory arises indirectly through the variations of $g_{\mu \nu }$. In contrast, in the new theory the Ricci scalar is interpreted as a coupling term in the Lagrangian density, which couples the metric tensor $g_{\mu \nu }$ (i.e., the physical field or generalized coordinate) with the Ricci tensor $R^{\mu \nu }$ which determines the geometry of spacetime and is considered extremal, namely identified with ${\hat{R}}^{\mu \nu }$, that is, expressed in terms of the background metric tensor $\hat{g}\left(r\right)$. The basic implications is therefore that the set $\left\{x_{R},H_{R}\right\}$ can be naturally identified as possible candidate for the classical Hamiltonian structure of GR.

The same features indicated above, on the other hand, should be considered as an obvious requirement for the establishment of canonical quantization theory of gravity and, in particular, the formulation of a manifestly covariant theory of quantum gravity. And indeed the implementation of such a task, referred to as CQG-theory, has been recently successfully achieved [29,30,32,33,34]. In fact, CQG-theory represents a new, fertile and promising field of theoretical research in mathematical physics and quantum field theory.

Several questions, not treated in this paper, however, remain still open and are left to future investigations. One undoubtedly concerns the excruciating long issue whether, despite all difficulties and inconveniencies pointed out here, a physically meaningful quantum theory can still be based on ADM theory and more specifically on the Wheeler-DeWitt quantum wave equation.
